# Interactions between Aβ and Mutated Tau Lead to Polymorphism and Induce Aggregation of Aβ-Mutated Tau Oligomeric Complexes

**DOI:** 10.1371/journal.pone.0073303

**Published:** 2013-08-12

**Authors:** Yoav Raz, Yifat Miller

**Affiliations:** 1 Department of Chemistry, Ben‑Gurion University of the Negev, Beer-Sheva, Israel; 2 Ilse Katz Institute for Nanoscale Science and Technology, Ben‑Gurion University of the Negev, Beer-Sheva, Israel; Oak Ridge National Laboratory, United States of America

## Abstract

One of the main hallmarks of the fronto-temporal dementia with Parkinsonism linked to chromosome 17 (FTDP-17) is the accumulation of neurofibrillary tangles in the brain as an outcome of the aggregation of mutated tau protein. This process occurs due to a number of genetic mutations in the *MAPT* gene. One of these mutations is the ∆K280 mutation in the tau R2 repeat domain, which promotes the aggregation vis-à-vis that for the wild-type tau. Experimental studies have shown that in Alzheimer’s disease Aβ peptide forms aggregates both with itself and with wild-type tau. By analogy, in FTDP-17, it is likely that there are interactions between Aβ and mutated tau, but the molecular mechanisms underlying such interactions remain to be elucidated. Thus, to investigate the interactions between Aβ and mutated tau, we constructed fourteen ∆K280 mutated tau-Aβ_17-42_ oligomeric complexes. In seven of the mutated tau-Aβ_17-42_ oligoemric complexes the mutated tau oligomers exhibited hydrophobic interactions in their core domain, and in the other seven mutated tau-Aβ_17-42_ oligoemric complexes the mutated tau oligomers exhibited salt-bridge interactions in their core domain. We considered two types of interactions between mutated tau oligomers and Aβ oligomers: interactions of one monomer of the Aβ oligomer with one monomer of the mutated tau oligomer to form a single-layer conformation, and interactions of the entire Aβ oligomer with the entire mutated tau oligomer to form a double-layer conformation. We also considered parallel arrangements of Aβ trimers alternating with mutated tau trimers in a single-layer conformation. Our results demonstrate that in the interactions of Aβ and mutated tau oligomers, polymorphic mutated tau-Aβ_17-42_ oligomeric complexes were observed, with a slight preference for the double-layer conformation. Aβ trimers alternating with mutated tau trimers constituted a structurally stable confined β-structure, albeit one that was energetically less stable than all the other constructed models.

## Introduction

Alzheimer’s disease (AD) is characterized by both senile plaques [1] and neurofibrillary tangles (NFTs) in the brain [2,3]. The senile plaques, which are extracellular deposits around nerve endings, are primarily made up of aggregates comprising oligomers and fibrils of Aβ peptide. NFTs, in contrast, are intracellular lesions that consist of aggregates of tau oligomers. Despite extensive studies of tau aggregates, the detailed structures of tau oligomers and tau fibrils are still to be fully elucidated. It is, however, known that the longest human tau isoform, hTau40 – observed in the central nervous system – consists of 441 residues. This isoform contains several domains, including the proline rich flanking regions P1 and P2 (residues 151-244) and four repeats R1-R4 (residues 244-369). These four repeats constitute the microtubule-binding domain that has both the^275^ VQIINK^280^ and the^306^ VQIVYK^311^ hexapeptide motifs, which have a propensity to adopt β-structures [4-7]. Atomic force microscopy (AFM) [8], Fourier transform infra-red (FT-IR) [9], circular dichroism (CD) [10] and X-ray fiber diffraction [11] experiments have shown that fragments of the β-structure in these repeats can interact with themselves and with one another to aggregate stably into fibrils [12-17]. *In vitro* studies have also shown the formation of Aβ-tau aggregates [18], and, most importantly, *in vivo* studies have shown that in AD, Aβ and tau are both present in the mitochondria, where they aggregate to form complexes of Aβ-tau [19-23]. Recently, Miller et al. [24] simulated the interactions of Aβ oligomers with oligomers of wild-type tau repeats R2, R3 and R4 and showed that the R2 repeat forms the most stable β-structure with Aβ [24].

The accumulation of the tau protein within the neuronal cells in AD differs from that in fronto-temporal dementia with Parkinsonism linked to chromosome 17 (FTDP-17), in which the accumulation of tau is caused by a number of mutations in the *MAPT* gene [3,25,26]. One of the best known mutations in FTDP-17 is the deletion ∆K280 mutation [27-29], which is located in the tau R2 repeat domain (residues 275-300) [30,31]. This mutation has been shown to promote the aggregation of the tau protein by enabling it to better adopt a β-structure vis-à-vis wild-type tau [14,32]. In analogy with the Aβ–tau aggregation in the mitochondria in AD, it is likely that in the FTDP-17 disease Aβ and mutated tau protein aggregate to form Aβ-mutated tau complexes.

To understand the mechanism through which different mutated tau-Aβ complexes may co-exist in FTDP-17 pathology, the interactions between Aβ and mutated tau should be probed. Experimental studies have shown that tau aggregation occurs via interactions between β-strand fragments in the tau protein and that tau interacts with Aβ [4-7], but how they interact and whether they interact via β-strands are still not known. Since all amyloidogenic peptides, including the tau protein, exhibit similar properties in that they polymerize into fibrils and share similar intermediates, it is conceivable that they may interact with these β-strand domains via a similar fibril formation mechanism. Previously, we simulated the specific interactions of Aβ with wild-type tau R2 via interactions of β-strand fragments of Aβ with β-strand fragments of tau repeats [24]. Here, we examined the interactions between the mutated ∆K280 tau repeat R2 and Aβ by constructing Aβ-mutated tau R2 repeat oligomeric complexes. We constructed two different structures of mutated ∆K280 tau repeat R2 oligomers, based on wild-type tau repeat R2 oligomers [Raz et al, in preparation]. One structure of mutated tau oligomers exhibited hydrophobic interactions in the core domain, and the other structure exhibited salt-bridge interactions in the core domain. We then combined each mutated tau repeat R2 oligomer with Aβ_17-42_ to investigate the preferred interactions between mutated tau repeat R2 oligomers and Aβ_17-42_ oligomers. We considered two types of interactions between mutated tau repeat R2 oligomers and Aβ_17-42_ oligomers: interactions of one monomer of the Aβ oligomer with one monomer of the mutated tau oligomer to form a single-layer conformation, and interactions of the entire Aβ oligomer with the entire mutated tau oligomer to form a double-layer conformation. Our simulations showed that the mutated tau-Aβ_17-42_ oligomeric complex has a rugged landscape that is characterized by various stable conformations with a preference to form double-layer conformations. We thus propose that the preferred mechanisms of the interactions between Aβ_17‑42_ oligomers and mutated tau repeat R2 oligomers occur via interactions of a single-layer of Aβ_17‑42_ oligomers and a single-layer of mutated tau repeat R2 oligomers to form a double-layer conformation along the fibril axis. Therefore, our study provides insight into the mechanisms though which mutated tau and Aβ interact to form aggregates. We thus believe that it can pave the way to future therapeutic efforts for the synthesis of novel drugs for FTDP-17, AD and other tauopathies that aim to prevent interactions between mutated tau protein and Aβ.

## Materials and Methods

### 1: Molecular dynamics (MD) simulations protocol

We constructed models of Aβ_17-42_, mutated ∆K280 tau oligomers and mutated tau-Aβ_17-42_ oligomers by using Accelerys Discovery Studio software (http://accelrys.com/products/discovery-studio/). MD simulations of the solvated oligomers were performed in the NPT ensemble using the NAMD [33] with the CHARMM27 force field [34,35]. The oligomers were energy minimized and explicitly solvated in a TIP3P water box [36,37] with a minimum distance of 15 Å from each edge of the box. Each water molecule within 2.5 Å of the oligomers was removed. Counterions were added at random locations to neutralize the oligomers’ charge. The Langevin piston method [33,38,39] with a decay period of 100 fs and a damping time of 50 fs was used to maintain a constant pressure of 1 atm. A temperature of 330 K was controlled by a Langevin thermostat with a damping coefficient of 10 ps^-1^ [33]. The short-range van der Waals interactions were calculated using the switching function, with a twin range cut-off of 10.0 and 12.0 Å. Long-range electrostatic interactions were calculated using the particle mesh Ewald method with a cutoff of 12.0 Å [40,41]. The equations of motion were integrated using the leapfrog integrator with a step of 1 fs. The solvated systems were energy minimized for 2000 conjugated gradient steps, where the hydrogen bonding distance between the β-sheets in each oligomer was fixed in the range 2.2-2.5 Å. The counterions and water molecules were allowed to move. The hydrogen atoms were constrained to the equilibrium bond using the SHAKE algorithm [42]. The minimized solvated systems were energy minimized for 5000 additional conjugate gradient steps and 20,000 heating steps at 250 K, with all atoms being allowed to move. Then, the system was heated from 250 to 330 K for 300 ps and equilibrated at 330 K for 300 ps. All simulations were run for 30 ns at 330 K. These conditions were applied to all of the examined structures.

### 2: Analysis details

We examined the structural stability of the studied oligomers by following the changes in the number of the hydrogen bonds between β-strands, with the hydrogen bond cut-off being set to 2.5 Å. This examination was performed by following the root-mean square deviations (RMSDs) and by monitoring the change in the inter-sheet distance (Cα backbone-backbone distance) in the core domain of all of the oligomers. In all the models that we constructed, the core domain of the mutated tau was defined as the distance between residue 280 and residue 293, and that for Aβ_17‑42_ as the distance between residue 22 and residue 35. We further investigated the averaged number of water molecules around each side-chain Cβ carbon within 4 Å for the mutated tau oligomers and for the mutated tau-Aβ_17‑42_ oligomers.

### 3: Generalized Born method with molecular volume (GBMV)

To obtain the relative conformational energies of mutated tau, Aβ_17-42_, and mutated tau-Aβ_17-42_ oligomers, the oligomer trajectories of the last 5 ns were first extracted from the explicit MD simulation excluding the water molecules. The solvation energies of all systems were calculated using the GBMV. In the GBMV calculations, the dielectric constant of water was set to 80. The hydrophobic solvent-accessible surface area (SASA) term factor was set to 0.00592 kcal/(mol Å^2^). Each conformer was minimized using 1000 cycles, and the conformational energy was evaluated by grid-based GBMV.

A total of 7000 conformations (500 conformations for each of the 14 examined conformers) were used to construct the energy landscape of the mutated tau-Aβ_17-42_ oligomers and to evaluate the conformer probabilities by using Monte Carlo (MC) simulations. In the first step, one conformation of conformer i and one conformation of conformer j were randomly selected. Then, the Boltzmann factor was computed as e^‑(Ej ‑ Ei)/KT^, where E_i_ and E_j_ are the conformational energies evaluated using the GBMV calculations for conformations i and j, respectively, K is the Boltzmann constant and T is the absolute temperature (298 K used here). If the value of the Boltzmann factor was larger than the random number, then the move from conformation i to conformation j was allowed. After 1 million steps, the conformations ‘visited’ for each conformer were counted. Finally, the relative probability of conformer n was evaluated as P_n_ = N_n_/N_total_, where P_n_ is the population of conformer n, N_n_ is the total number of conformations visited for the conformer n, and N_total_ is the total steps. The advantages of using MC simulations to estimate conformer probability lie in their good numerical stability and the control that they allow of transition probabilities among several conformers.

Using all 14 conformers and 7000 conformations (500 for each conformer) generated from the MD simulations, we estimated the overall stability and populations for each conformer based on the MD simulations, with the energy landscape being computed with GBMV for all conformers. For the complex kinetics of amyloid formation, the group of the 14 conformers is likely to represent only a very small percentage of the ensemble. Nevertheless, the carefully selected models cover the most likely structures.

We further examined the energy landscape computed with GBMV for all conformers by comparing the results to integrated fitted Gaussians obtained using Origin Pro 8 (see Text S1 and Figures S1-S4). 

### 4: Reaction coordinates for the formation of Aβ-mutated tau oligomers from Aβ and mutated tau oligomers

To investigate the stability of each soluble Aβ-mutated tau oligomer, the conformational energies were computed for Aβ oligomers, mutated tau oligomers, and Aβ-mutated tau oligomers (Tables S1-S3). The conformational energies for each oligomer are based on the energy computed with the GBMV method. For each oligomer, a total of 500 conformations from the last 5 ns of the simulations were used to evaluate the conformational energy.

We estimated the relative stability of each Aβ-mutated tau oligomer by comparing its energy with the energies of the two separate components, Aβ and mutated tau oligomers, as illustrated by the following chemical “reaction”:

$${\left(A\beta \right)}_{n}+{\left(mutated-Tau\right)}_{n}\Leftrightarrow {\left(A\beta \right)}_{n}•{\left(mutated-Tau\right)}_{n}$$ (1)

where n indicates the number of monomers within an oligomer. In the current study n = 6.

## Results and Discussion

### 1: Constructed models of mutated tau hexamers

**Figure 1:**
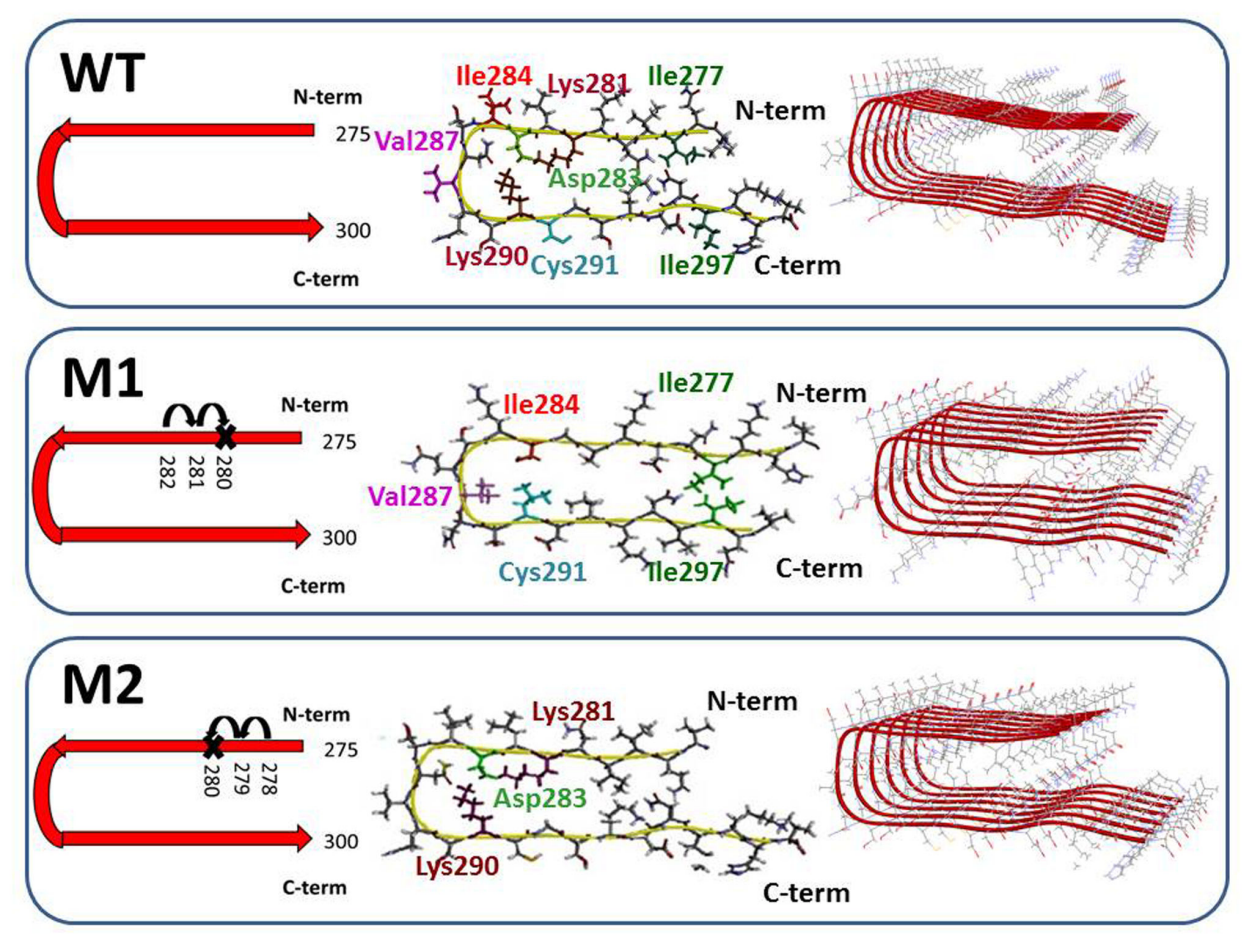
Schematic representations of molecular structures of the wild-type and the two constructed models of the mutated ∆K280 tau repeat R2 monomers (275-300). In M1, the ΔK280 deletion mutation was obtained by ‘shifting’ the N-terminal sequence towards K280. This model is stabilized by hydrophobic interactions in the core domain. In M2, the ΔK280 deletion mutation was obtained by ‘shifting’ the C-terminal sequence towards K280. This model is stabilized by salt-bridge interactions in the core domain. Models M1 and M2 were constructed as hexamers.

To construct the mutated tau ∆K280 repeat R2 oligomers, we applied the previously constructed model of the tau repeat R2 oligomer [24,34], in which the backbone hairpins were based on the Lührs ssNMR model of Aβ_17-42_ [43]. Two possible models were constructed to form the mutated tau ∆K280 repeat R2 oligomers on the basis of the wild-type tau repeat R2 (Figure 1). In the first model, designated M1, the deletion mutation was obtained by ‘shifting’ the sequence from the C-terminal end towards the ΔK280 deletion site (as seen in Figure 1), while in the second model, M2, the deletion mutation was obtained via ‘shifting’ the N-terminal sequence towards the ΔK280 mutation site (as seen in Figure 1). Model M1 is characterized by hydrophobic interactions (between Ile277 and Ile297 and between Leu284 and Val287) in the core domain of the oligomer. In contrast, model M2 is characterized by salt-bridge interactions (between Lys281, Asp283 and Lys290) in the core domain of the oligomer. Since previous computational modeling studies have shown that hexamers are the minimal oligomeric size of amyloid to adopt a fibril-like structure [44,45], we sought to keep the number of structural models manageable with respect to computational power and timescale by constructing hexamers.

### 2: Constructed models of mutated tau-Aβ dodecamers

**Figure 2:**
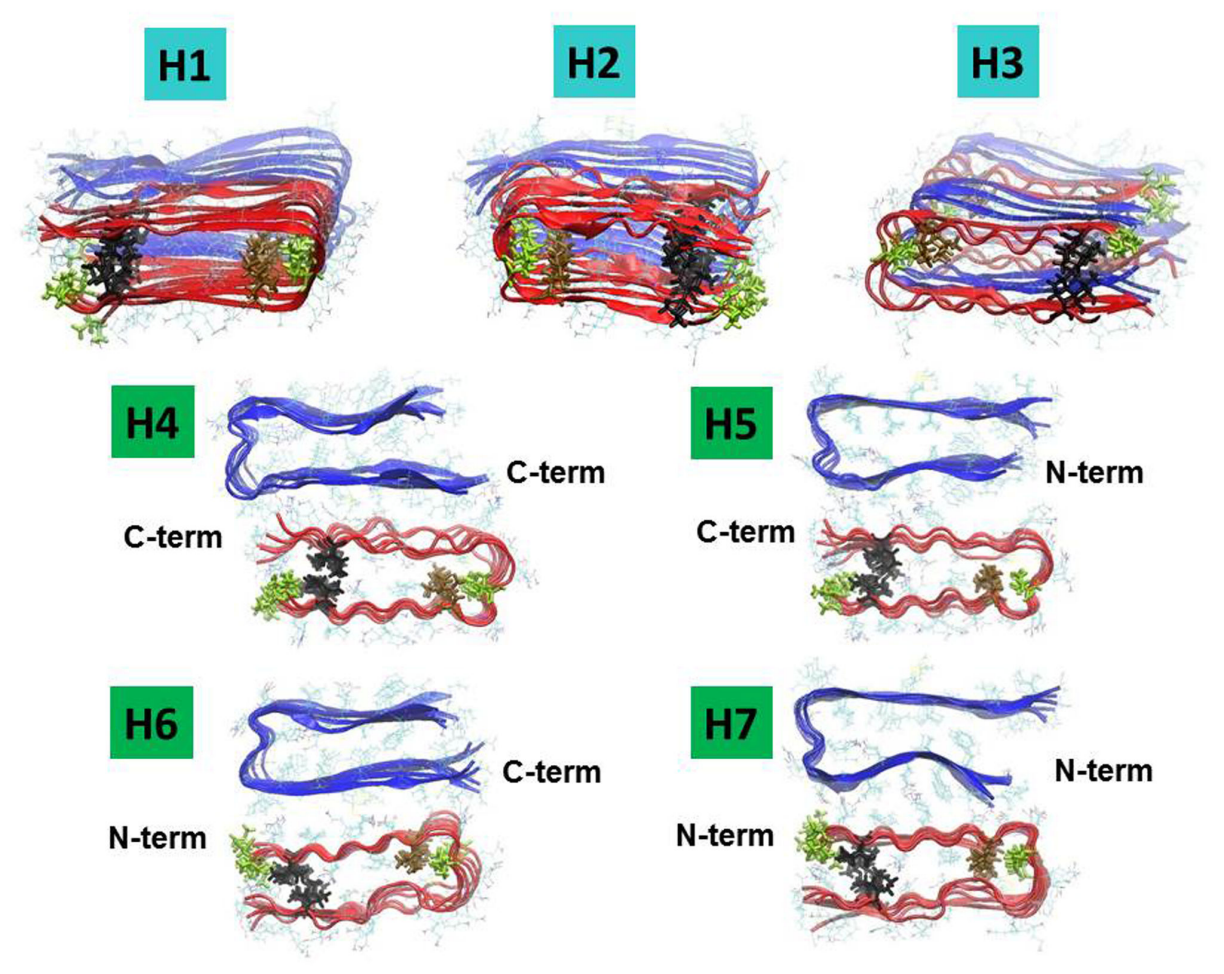
Constructed models of oligomeric complexes of mutated ∆K280 tau repeat R2-Aβ_17-42_. The mutated ∆K280 tau repeat-R2 oligomers are based on model M1 in which hydrophobic interactions stabilize the core domain of the β-hairpins (Figure 1). Aβ_17-42_ oligomers (blue) and the mutated ∆K280 tau repeat R2 oligomers (red) are organized in parallel orientation. Residue colors: Cys (brown), Ile (black), Val (green). Models H1, H2 and H3 represent single-layer conformations. In H1 and H2, the oligomers are arranged in parallel and antiparallel orientation, respectively. In H3, trimers of Aβ_17-42_ and trimers of mutated ∆K280 tau repeat R2 are alternated in parallel orientation. Models H4-H7 offer four possible arrangements of double-layer conformations that differ in the interactions between C-terminus domains/N-terminus domains of each oligomer to form double-layer conformations.

**Figure 3:**
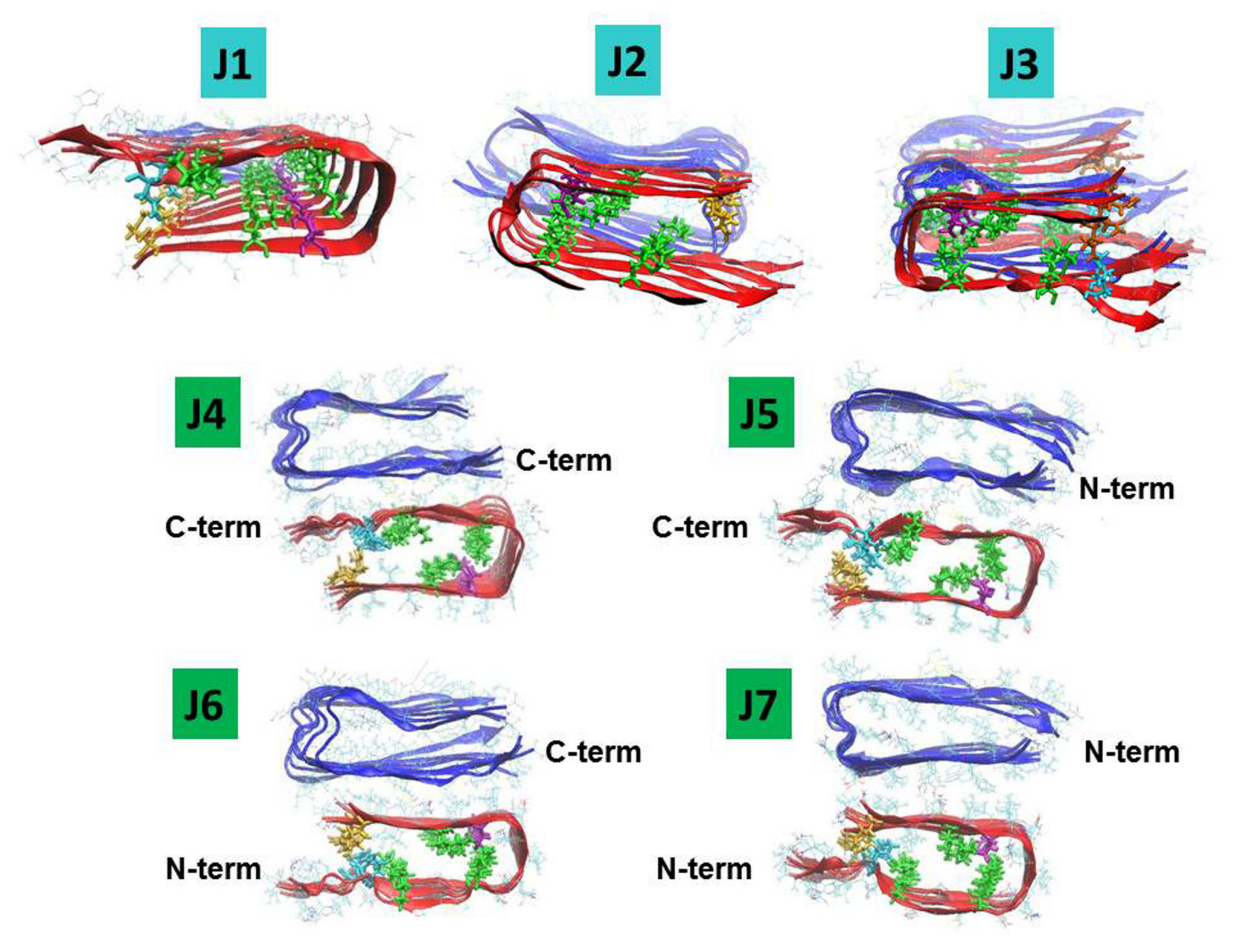
Constructed models of the oligomeric complexes of mutated ∆K280 tau repeat R2-Aβ_17-42_. The mutated ∆K280 tau repeat R2 oligomers are based on model M2 in which salt-bridge interactions stabilize the core domain of the β-hairpin (Figure 1). Aβ_17-42_ oligomers (blue) and the mutated ∆K280 tau repeat R2 oligomers (red) are organized in parallel orientation. Residue colors: Lys (green), Asp (purple), Asn (cyan), Gln (orange). Models J1, J2 and J3 represent single-layer conformations. In J1 and J2, the oligomers are arranged in parallel and antiparallel orientations, respectively. In J3, trimers of Aβ_17-42_ and trimers of mutated ∆K280 tau repeat R2 are alternated in parallel orientation. Models J4-J7 represent four possible arrangements of double-layer conformations that differ in the interactions between C-terminus domains/N-terminus domains of each oligomer to form double-layer conformations.

Since it has been proposed that the parallel orientation is preferred for Aβ_17-42_ oligomers [43,46], we considered only hexamers with parallel organization when constructing Aβ_17‑42_ oligomers. We associated each one of the two mutated hexamers (models M1 and M2) with the Aβ_17‑42_ hexamer and considered ensembles of various alignments between them. The constructed mutated tau M1-Aβ_17‑42_ dodecamers are illustrated in Figure 2 (models H1–H7), and the constructed mutated tau M2-Aβ_17‑42_ dodecamers are illustrated in Figure 3 (models J1–J6).

Models H1–H3 and models J1–J3 show interactions between the β-strands in the termini of the hairpins of mutated tau and the β-strands in the termini of the hairpins of Aβ17‑42 forming single-layer conformations with various organizations between the hairpins of Aβ and the hairpins of mutated tau. Since trimers of Aβ are known to be toxic species [47-49], we also considered parallel arrangements of Aβ trimers alternating with mutated tau trimers, as seen in models H3 and J3.

Models H4–H7 and models J4–J7 show interactions between all the β-strands of the C-termini or the N-termini of Aβ_17‑42_ hexamers with all the β-strands of the C-termini or the N-termini of mutated tau hexamers to form double-layer conformations. Previous studies have reported double-layer conformations for other fragments of Aβ, such as Aβ_16-21_, Aβ_27-32_ [50], Aβ_17‑42_ [51], Aβ_9‑40_ [52], and Aβ_1‑40_ [53], and for other amyloids, such as amylin [54], β_2_-microglobulin [55], and Sup35p [56]. In our study, we examined the double-layer conformations that are formed via the interactions between Aβ_17‑42_ oligomers and mutated tau oligomers.

### 3: Polymorphic mutated tau-Aβ_17-42_ oligomers play a role in the fibrilization of tau-Aβ_17-42_

One of the most difficult aspects of protein aggregation – and indeed amyloid aggregation – to investigate is aggregate polymorphism [12,46,52,57-59]. In principle, there are thousands of possible patterns of intra- and inter-residue amyloid fibril backbone β-strand organizations for in-register and out-of-register interactions. Moreover, there are also in-register alignments with double-layer β-strand segment arrangements within and between sheets. In interactions between two different types of amyloids even more possible polymorphic states can be expected.

**Figure 4:**
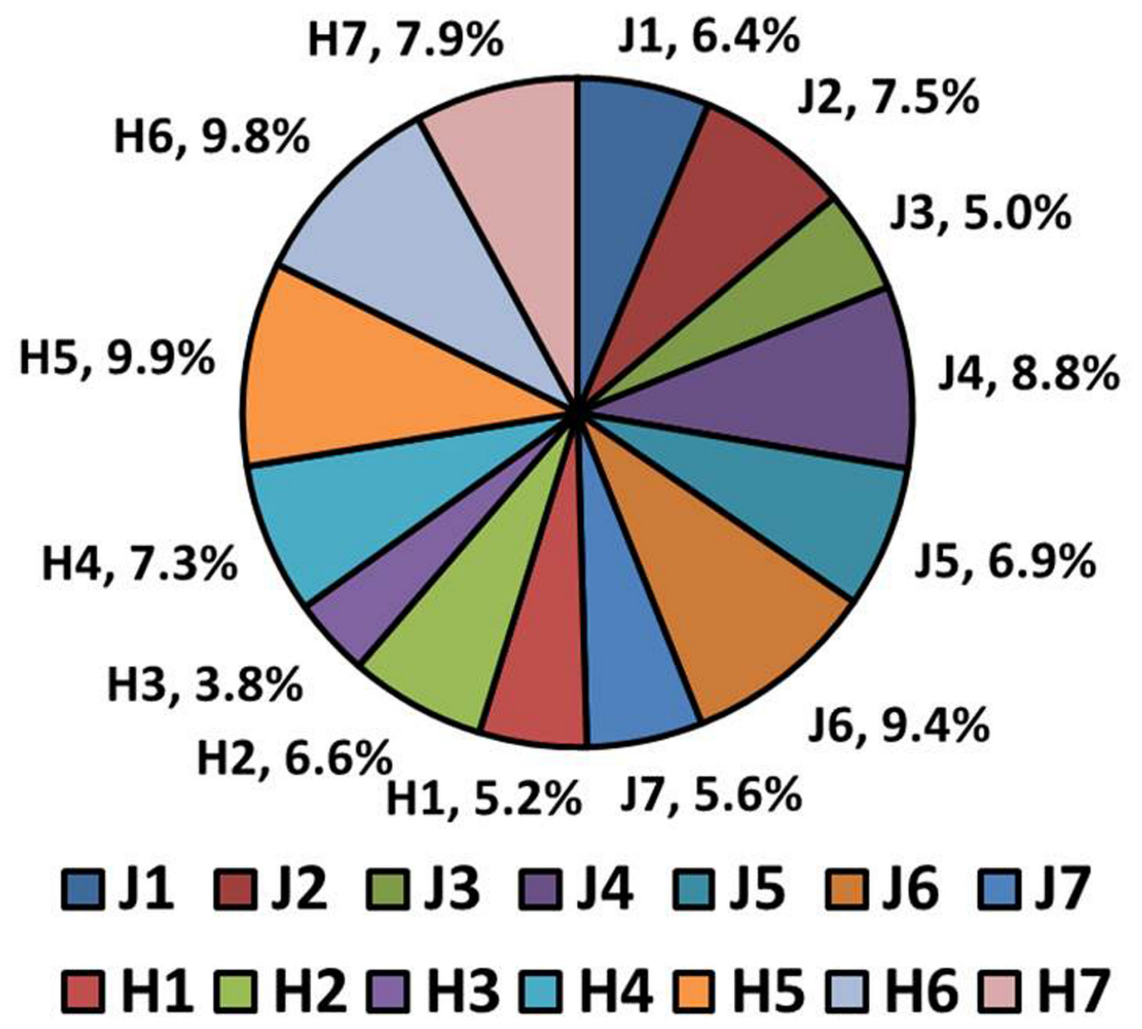
Calculated populations of the constructed models using the GBMV method [78,79] and Monte-Carlo simulations.

To investigate the polymorphic states of our models of mutated tau-Aβ_17-42_ oligomers, we applied the GBMV method to compute the relative conformational energies of all constructed models and MC simulations to estimate the overall populations for each model. As may be seen from the computed energies (Table S1) and the population results (Figure 4), some of the models H1–H7 and J1–J7 exhibit similar stabilities and similar populations; for example, each of the models H5, H6 and J6 comprises ~10% of the population, while each of the models H1, J3 and J7 comprises ~5% of the population. These results indicate a rugged landscape of mutated tau-Aβ_17‑42_ oligomers. Polymorphic states may be derived from differences in three major structural features: backbone conformation, backbone orientation, and in the way in which oligomers with almost identical structures associate with one another [60]. Previously, Miller et al. [46] illustrated polymorphism of the Aβ_1-40_ oligomer, which derived from the N-terminal arrangement and the U-turn shape of the threefold symmetry based on the models suggested by Tycko and coworkers [61] and by Lührs et al. [43]. However, for the tau repeats, only the Lührs model demonstrated stable structures [24]. In this study, we therefore applied the Lührs model to construct mutated tau oligomers. We showed that the mutated tau-Aβ_17-42_ oligomers differ both in the backbone orientation between the Aβ_17‑42_ oligomers and the mutated tau oligomers (i.e., parallel and antiparallel orientations) and in the way which Aβ_17‑42_ oligomers and mutated tau oligomers associate (i.e., interactions between Aβ_17‑42_ oligomers and mutated tau oligomers to form single-layer conformations or double-layer conformations). In addition, in this study we considered two possible polymorphic states of mutated tau oligomers, M1 and M2, differing in backbone conformations, that interacted with Aβ_17-42_ oligomers. Our simulations did indeed reveal polymorphic states; for example, although models J1 and J5, which are both based on model M2, exhibited similar stabilities and similar populations, in model J1 the Aβ_17‑42_ oligomer associated with M2 to form a single-layer conformation with a parallel orientation, while in model J5 the Aβ_17‑42_ oligomer associated with M2 to form a double-layer conformation. In another example, models H6 and J6 exhibited similar stabilities and similar populations, and although model H6 is based on model M1 and model J6 is based on model M2, the mutated tau oligomers associated with Aβ_17‑42_ oligomers to form double-layer conformations in both models.

Finally, it was previously shown that the parallel orientation is strongly preferred in Aβ_17‑42_ oligomers [46], but in our study there was a slight preference for the antiparallel arrangement between Aβ_17‑42_ oligomers and mutated tau oligomers.

### 4: Mutated tau oligomers show a slight preference for associating with Aβ_17-42_ oligomers to form double layer conformations

One of the most important questions in the study of the interactions between mutated tau oligomers and Aβ_17‑42_ oligomers is whether mutated tau oligomers interact with Aβ_17‑42_ oligomers to form single-layer or double-layer conformations. We examined this issue with the aim of gaining insight into the mechanism of the interactions between these two types of amyloids. It may be seen from Figure 4 that the constructed models produced population values in the range of ~4-10%. Despite the polymorphism of the mutated tau-Aβ oligomers, all models with double-layer conformations (excluding J7) each comprised the highest portion of the total possible conformations (above 7%) compared to the models with the single-layer conformations (with the exception of model J2, which showed above 7% of the populations). Moreover, the double-layer conformations H4-H7 and J4–J7 constituted a sum of ~66% of the total possible population. It should be note here that among all the single-layer conformations, J2 exhibited the highest conformational energy and comprised the highest population. The relatively high population of J2 may be due to the hydrophobic interactions between the two types of amyloids (Figure S5).

It was previously shown that the double-layer conformations in Aβ_17‑42_ oligomers exhibited relatively stable structures and higher populations than the single-layer conformations [46]. In this study, we also found that the double-layer conformations of the cross-amyloid mutated tau-Aβ_17‑42_ oligomers are slightly preferred over the single layer conformations. In the double-layer conformations, the number of contact interactions between two β-strands may increase the stability of the structures, while these interactions do not exist in single-layer conformations. Among the double-layer conformations in which the mutated tau adopts the M1 structure, model H5 comprises the highest population, and among the double-layer conformations in which the mutated tau adopts the M2 structure, model J6 comprises the highest population. Interestingly, while in M1 the core domain is characterized by hydrophobic interactions, in the double-layer conformations salt-bridge interactions stabilize the associations between M1 and the Aβ oligomer (Figure S6). In contrast, while in M2 the core domain is characterized by salt-bridge interactions, hydrophobic interactions stabilize the associations between M2 and Aβ oligomer (Figure S7). Model J7 comprised a relatively low population in comparison with the other double-layer conformations, indicating a lack of salt-bridge and hydrophobic interactions between the two types of amyloids in the double-layer conformation.

To analyze the structural stability of the most stable double-layer conformations, H5 and J6, we examined the percentage of hydrogen bonds and the inter-sheet distances between the β-strands along the MD simulations. One can see that the percentage of the hydrogen bonds that were maintained along the simulations for these two conformations was relatively high, i.e., above 80% (Figures S8 and S9). Interestingly, in the interactions of mutated tau and Aβ oligomers, the inter-sheet distances between the β-strands of Aβ oligomers is kept at ~13 Å in these two conformations, indicating stable structures (Figures S10 and S11). Furthermore, the RMSDs of these conformations confirm that these structures are stable (Figures S12 and S13). In summary, we propose that the mechanisms of the interactions between Aβ oligomers and mutated tau R2 repeat oligomers show a slight preference for interactions between the β-strands of the two amyloids to form double-layer conformations.

### 5: Interactions between Aβ trimers alternating with mutated tau trimers form similar inter-sheet distances in the fibril-like structure

Trimers and tetramers of Aβ are known to be relatively more toxic than oligomers of other sizes [47]. We therefore examined the interactions between trimers of Aβ alternating with trimers of mutated tau to form Aβ-mutated tau dodecamer structures. We tested the interactions of Aβ trimers with M1 by constructing model H3 and with M2 by constructing model J3. Both the H3 and J3 models comprise smaller populations and are relatively less energetically stable than the other ensembles of structures that we examined in this study. However, the percentage of hydrogen bonds in both models is around 70%, which indicates that H3 and J3 are structurally stable conformations. Interestingly, as may be seen in Table S2, in all the models other than H3 and J3 the inter-sheet distances for Aβ oligomers, mutated tau oligomers and Aβ-mutated tau oligomers differ dramatically within each model, whereas in models H3 and J3 the values of these distances are similar (for H3: 14-14.5 Å and for J3: 19-20 Å). Therefore, we propose that these structures are thermodynamically unfavorable even though they are structurally stable. Structurally, these two types of amyloidogenic small oligomers synergistically stabilize a fibril-like structure by maintaining a uniform diameter along the fibril’s axis.

### 6: Structural stability of Aβ-mutated tau single layer conformations

We also examined the structural stability of the single layer conformations H1, H2, J1 and J2 by following the changes in the number of hydrogen bonds between the β-strands in the β-hairpins (Figures S8 and S9), by monitoring the change in the inter-sheet distances in the core domain (Figures S10 and S11), and by measuring the RMSDs (Figures S12 and S13) during the MD simulations.

A fairly high portion of hydrogen bonding (>80%) was retained throughout the MD simulations for H1, H2, J1 and J2. We found (Figures S10 and S11) that while the inter-sheet distances in the mutated tau monomers and in Aβ monomers are relatively large, in the interface between one Aβ monomer and one mutated tau monomer the inter-sheet distance is relatively small, indicating a well-packed fibril-like formation for H1, H2, J1 and J2. Finally, the RMSDs of all Aβ-mutated tau single-layer conformations demonstrate stable structures (Figures S12 and S13).

### 7: Interactions between Aβ oligomers and mutated tau oligomers to form double-layer conformations are energetically favored due to an ‘exothermic reaction’

**Figure 5:**
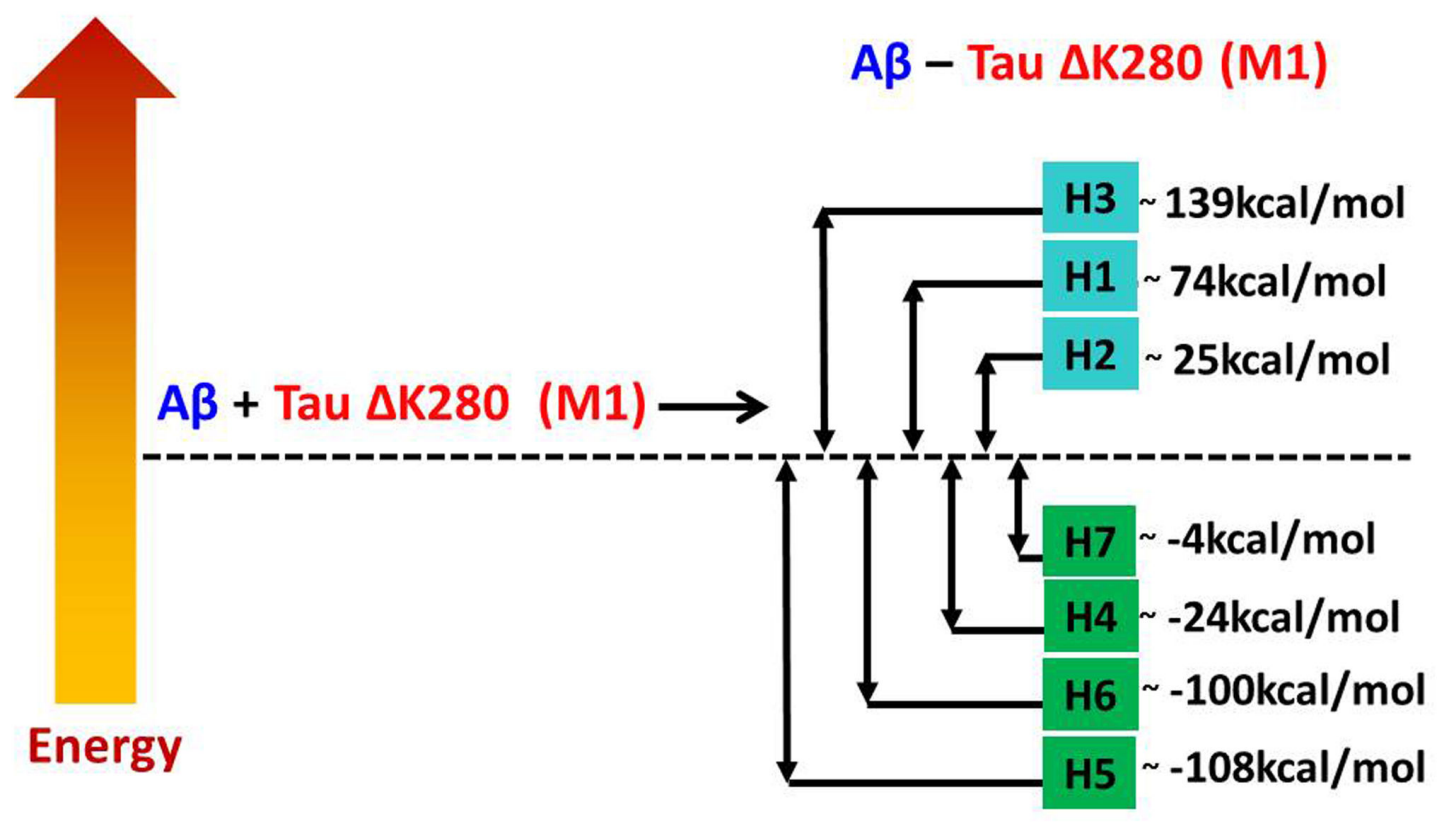
Relative energies of the two separate components Aβ_17-42_ and mutated tau M1 and of the Aβ_17-42_-mutated tau M1 complexes (H1-H7 models) computed by the GBMV method [78,79].

**Figure 6:**
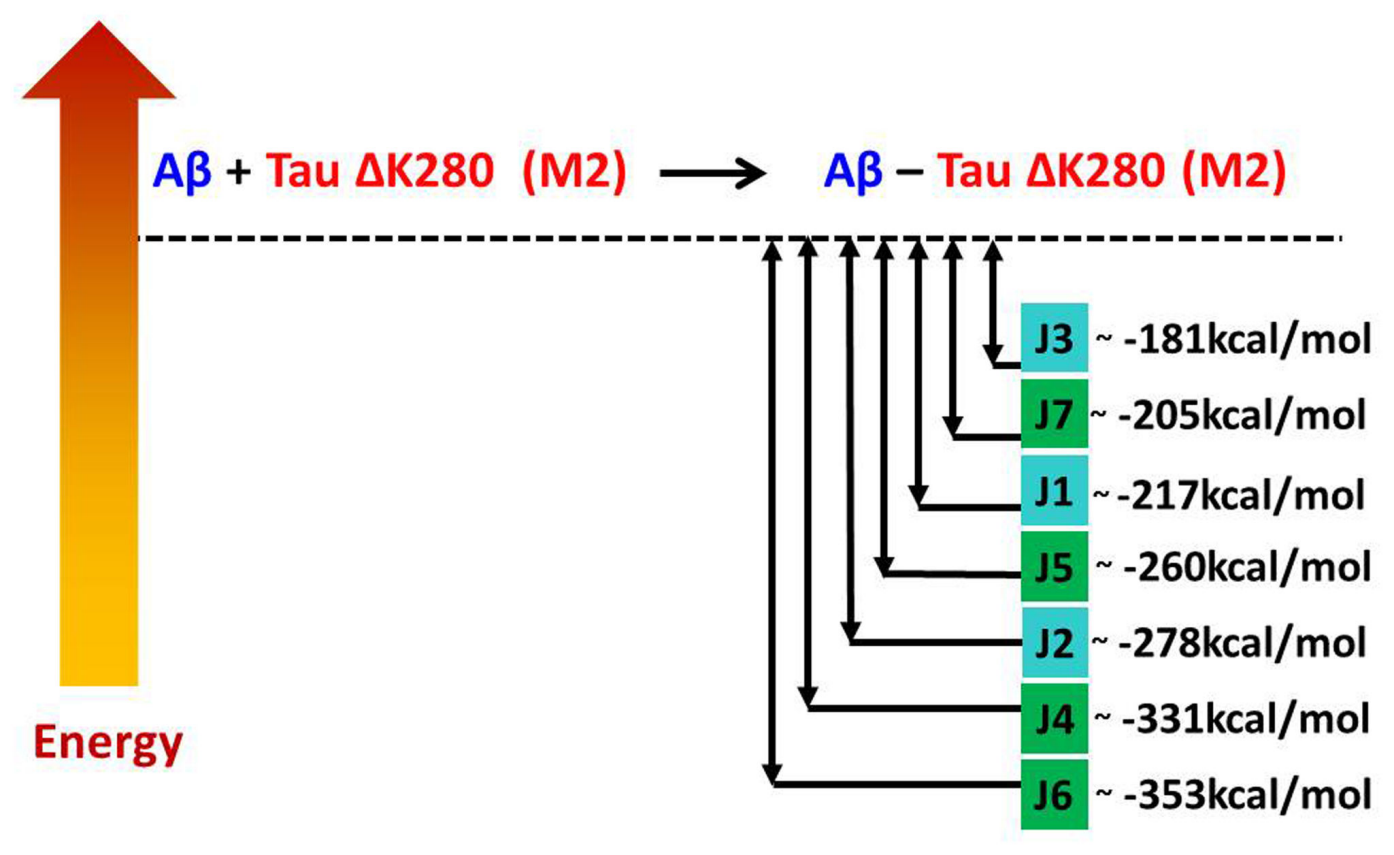
Relative energies of two separate components Aβ_17-42_ and mutated tau M2 and of the Aβ_17-42_-mutated tau M2 complexes (J1-J7 models) computed by the GBMV method [78,79].

To examine the effect of the interactions between Aβ and mutated tau, we estimated the relative stability of each Aβ_17-42_-mutated tau oligomer by comparing its energy with the energies of the two separate components, Aβ_17-42_ oligomers and mutated tau oligomers. Figures 5 and 6 illustrate the “reaction coordinates” for the formation of Aβ_17‑42_-mutated tau oligomers from Aβ_17‑42_ oligomers and mutated tau M1 and M2 oligomers, respectively. The relative conformational energies of Aβ_17‑42_ oligomers and mutated tau M1 and M2 oligomers are given in Table S3. The formation of all double-layer conformations of Aβ_17‑42_-mutated tau oligomers (H4-H7 and J4–J7) is energetically highly preferred due to the ‘exothermic reaction’ of formation. Interestingly, while all the single-layer conformations in which the mutated tau is in the M2 form (J1, J2 and J3) would be produced in an ‘exothermic reaction’, all the reactions leading to the single-layer conformations in which the mutated tau is in the M1 form (H1, H2 and H3) would be produced in ‘endothermic reaction’.

As noted above, some of the double-layer conformations comprise the highest populations among the ensembles of possible conformations that we examined in this study and some show similar populations. Interestingly, models H6 and J6, which exhibit similar structural features (i.e., the C-termini of the Aβ17-42 oligomers interact with the N-termini of mutated tau) have similar stabilities and similar populations (Figure 4 and Table S1), but in the “reaction coordinates” model J6 is dramatically more stabilized than model H6 (Figure 5 and Figure 6). In general, all the double-layer conformations of the J models appear to be more stabilized than those of the H models. In the double-layer conformations of the J models, hydrophobic interactions between Aβ17-42 oligomers and mutated tau oligomers stabilize the conformations, while in the double-layer conformations of H models, the salt-bridge interactions stabilize the conformations. Therefore, we propose that the hydrophobic interactions between these two types of amyloids strongly contribute to the fibrilization of the double-layer conformations.

### 8: Solvation of backbone residues in mutated tau and Aβ_17-42_-mutated tau oligomeric complexes indicate the stability of the complexes

We further examined the stability of the Aβ_17-42_-mutated tau oligomeric complexes by investigating the backbone solvation. To this end, we compared the backbone solvation of models H1–H7 with that of model M1 (Figure S14) and the backbone solvation of models J1–J7 with that of model M2 (Figure S15). The backbone solvation values for all residues were similar for models H1, H3–H7 and model M1. Model H2 and model M1 have similar backbone solvation values for all residues, except for residues Ile277, Leu282 and Leu284 (Figure S14). In model H2 the inter-sheet distances are relatively small, and thus these three residues are less solvated than in model M1. The single-layer conformation of model H2 exhibits intramolecular hydrophobic interactions between Ile277, Ile297 and Leu300 of mutated tau, and intermolecular hydrophobic interactions between Leu288 of mutated tau and Ala21 of Aβ_17‑42_ (Figure S16).

The backbone solvation values for all residues were similar for models J1–J7 and model M2, except for residue His299, which was less solvated in models J1–J7, since this residue is less exposed to the water and undergoes a stronger interaction with the Aβ_17‑42_ monomer in the single-layer conformations (models J1–J3). In addition, for the double-layer conformations the His299 has stronger interactions with Aβ_17-42_ oligomers in models J5 and J7.

## Summary and Conclusions

The aggregation of Aβ into amyloid plaques is characteristic of AD, while the aggregation of mutated tau into NFTs is a process long known to be related to FTDP-17. One of the most common mutations in FTDP-17 is the deletion mutation ΔK280, which is located in the R2 repeat domain of the tau protein. Recent experimental studies suggest that Aβ may interact directly or indirectly with tau to promote the formation of NFTs [19-22,62-68]. In light of the previous simulations of Miller et al. [24] showing that Aβ has a strong preference for interacting with the R2 repeat in the tau protein (rather than with repeats R1, R3 or R4) and of experimental studies showing that the mutation ΔK280 in the tau R2 repeat promotes aggregation (vis-à-vis wild-type tau R2 repeat) [3,14], we were motivated to investigate the mechanisms and the related interactions through which mutated tau and Aβ assemble to form preferred organizations of oligomeric complexes.

To this end, we applied all-atom MD simulations in an explicit solvent and constructed various models of Aβ-mutated tau oligomeric complexes. Our models were derived from previously constructed models for Aβ-tau oligomers that were based on experimental data; namely, the oligomers were based on the experimental model suggested by Lührs et al. [43], as previously studied by Miller et al. [24] for Aβ_17-42_, and on tau oligomer models that were also based on experimental data that showed a high propensity for a β-structure in the tau R2 repeat domain [9]. In the current study, we considered two different structural models for the ΔK280 mutation in the tau R2 repeat, the first model was characterized by hydrophobic interactions in the core domain (M1) and the second model, by salt-bridge interactions in the core domain (M2).

Three main conclusions may be drawn from our simulations: First, mutated tau-Aβ oligomeric complexes are polymorphic in their gross organizations. Since mutated tau oligomers are polymorphic and Aβ oligomers also polymorphic, mutated tau-Aβ oligomeric complexes are also expected to be polymorphic. Most of the mutated tau-Aβ oligomeric complexes that were investigated in this study were thermodynamically preferred over the separate mutated tau oligomers and Aβ oligomers. Therefore, we propose that mutated tau oligomers and Aβ oligomers synergistically stabilize each other, leading to polymorphic states. The synergistic stabilization was confirmed by the formation of well-defined β-sheet structures for both Aβ oligomers and mutated tau oligomers along the fibril-like axis. Second, among the polymorphic mutated tau–Aβ oligomeric complexes, the double-layer conformations were slightly preferred, indicating that the preferred mechanism for the interactions between these two types of amyloids takes place via the formation of double-layer conformations. Since the topology of the single-layer conformations differs from that of the double-layer conformations, these topologies may be explored experimentally in the future. Third, interactions between trimers of Aβ alternating with trimers of mutated tau form oligomeric complexes that are thermodynamically less preferred, but structurally Aβ trimers and mutated tau trimers stabilize each other to form similar inter-sheet distances in the core domain along the fibril axis.

The hydrophobic regions in the tau R2 repeat interact with microtubules and stabilize them. Mutations in the tau R2 repeat lead to decreased binding affinity of tau to the microtubules [3] and hence to accelerated mutated tau aggregation [3,69-73]. Some of the mutations may weaken tau’s ability to bind to microtubules [74-76] and thus may cause degradation of the microtubules, leading to neurotoxicity. A recent study [77] indicated that Aβ oligomers induce the aggregation of tau and the formation of β-sheet rich neurotoxic tau oligomers. In addition, since Aβ trimers which are known to be toxic species [47-49], we propose in this study that Aβ trimers may stabilize the formation of β-sheets in the mutated tau R2 repeat trimers, even though such a conformation is energetically less favorable. Yet, the mechanism for the increased toxicity is still controversial and further investigations should be performed.

## Supporting Information

Text S1 **Analysis of populations for Aβ-mutated tau models.**
(PDF)

Figure S1 **Scatter charts of the 500 conformations for H1-H4: obtained from the GBMV energy values extracted from the last 5 ns of each model: H1-H4 (black squares).** The scatter charts represent the “histograms” of the number of conformations in energies’ ranges. The fitted curves (red line) were computed directly by Origin Pro 8.
(TIF)

Figure S2 **Scatter charts of the 500 conformations for J1-J4: obtained from the GBMV energy values extracted from the last 5 ns of each model: J1-J4 (black squares).** The scatter charts represent the “histograms” of the number of conformations in the energy ranges. The fitted curves (red line) were computed directly by Origin Pro 8.
(TIF)

Figure S3 **Comparison between GBMV and fitted Gaussian for H1-H7: Comparison between the averaged energies of the 500 conformations using the GBMV method and the energies obtained from the primary peaks of models H1-H7 using the peak fitting function of the origin with standard deviations **(**Figure S13**).
(TIF)

Figure S4 **Comparison between GBMV and fitted Gaussian for J1-J7: Comparison between the averaged energies of the 500 conformations using the GBMV method and the energies obtained from the primary peaks of models J1-J7 using the peak fitting function of the origin with standard deviations **(**Figure S14**).
(TIF)

Figure S5 **Hydrophobic interactions in model M2: Model J2 constitutes a relatively high portion of the total population in comparison with the other single-layer conformations, probably due to the hydrophobic interactions between Aβ_17-42_ (blue) and mutated tau (red) oligomers, which stabilized the structure.**
(TIF)

Figure S6 **Salt-bridge interactions in model H5: The double-layer conformation of model H5 is stabilized by salt-bridge interactions in the interface region between Aβ_17-42_ (blue) and mutated tau (red) oligomers.**
(TIF)

Figure S7 **Hydrophobic interactions in model J6: The double-layer conformation of model J6 is stabilized by hydrophobic interactions in the interface region between Aβ_17-42_ (blue) and mutated tau (red) oligomers.**
(TIF)

Figure S8 **The fraction of the number of hydrogen bonds in models H1-H7: The fraction of the number of hydrogen bonds (in percentage) between all β-strands compared to the number in the initial oligomer for models H1-H7.**
(TIF)

Figure S9 **The fraction of the number of hydrogen bonds in models J1-J7: The fraction of the number of hydrogen bonds (in percentage) between all β-strands compare to the number in the initial oligomer for models J1-J7.**
(TIF)

Figure S10 **The averaged inter-sheet (Cα backbone-backbone) distances for models H1-H7 along the molecular dynamics (MD) simulations**.
(TIF)

Figure S11 **The averaged inter-sheet (Cα backbone-backbone) distances for models J1-J7 along the molecular dynamics (MD) simulations.**
(TIF)

Figure S12 **RMSDs of H1-H7.**
(TIF)

Figure S13 **RMSDs of J1-J7.**
(TIF)

Figure S14 **The average number of water molecules around each side chain Cβ carbon (within 4 Å) for models H1-H7.**
(TIF)

Figure S15 **The average number of water molecules around each side chain Cβ carbon (within 4 Å) for models J1-J7.**
(TIF)

Figure S16 **Hydrophobic interactions in model H2: The single-layer conformation of model H2 illustrates intramolecular hydrophobic interactions between Ile277, Ile297 and Leu300 of mutated tau, and intermolecular hydrophobic interactions between Leu288 of mutated tau and Ala21 of Aβ_17-42_.**
(TIF)

Table S1 **The conformational energies (computed using the GBMV calculations) and the populations of models H1-H7 and model J1-J7.**
(PDF)

Table S2 **The averaged inter-sheet (Cα backbone-backbone) distances measured in the last 5 ns of the MD simulations for models H1-H7 and models J1-J7.**
(PDF)

Table S3 **The conformational energies (computed using the GBMV calculations) and the populations of wild-type (WT) TauR2 repeat oligomer, the mutated tau M1 and M2 models and Aβ_17-42_ oligomer.**
(PDF)
