# Supplementary material for: Interactions between Aβ and Mutated Tau Lead to Polymorphism and Induce Aggregation of Aβ-Mutated Tau Oligomeric Complexes
Source: PLoS One. 2013 Aug 12;8(8):e73303. doi: 10.1371/journal.pone.0073303 (PMC3741189; doi:10.1371/journal.pone.0073303)
Supplement: Text S1 — Analysis of populations for Aβ-mutated tau models. (PDF) [file pone.0073303.s001.pdf]

Text S1: Analysis of populations for Aβ-mutated tau models

To compare the results of the relative conformational energies obtained from th8e GBMV method with another statistical method, we used Origin Pro 8 to analyze the distribution of the conformational energies. All 500 values for each model extracted from the last 5 ns of the simulations were used to characterize the distribution by histograms (seen in black squares in Figures S13 and S14). Two peaks were chosen to fit a Gaussian function for each peak for all models. All models showed only one peak, with the exception J2, for which two peaks were observed. For J2, the main peak represents the main population, while the smaller peak (or the tail) represents the smallest population. This finding explains the unexpectedly high population of J2 versus J1. The fitted Gaussians were integrated with Origin Pro 8.

The Gaussian function was defined as follows:

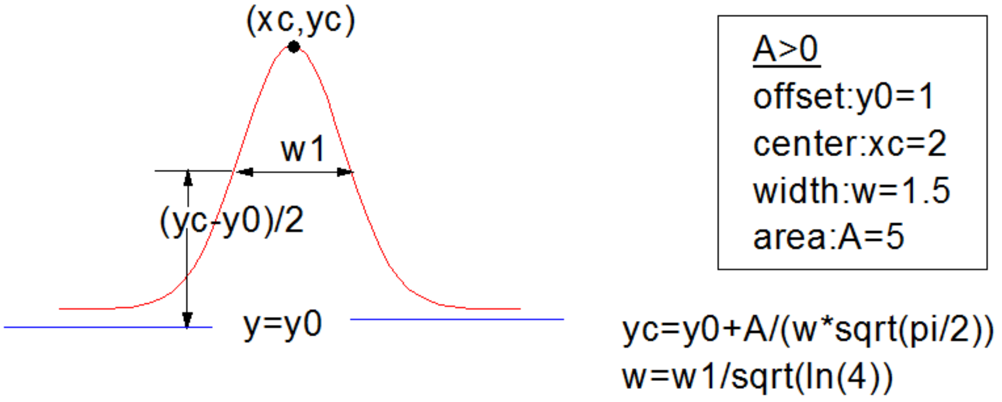

$$y = y_0 + \frac{A}{w\sqrt{\pi/2}} e^{-2\frac{(x-x_c)^2}{w^2}}$$

where X_c is the median value, A is the amplitude, w is the width and y_0 are the initial Cartesian coordinates.

Since there was some overlap of the peaks, we compared the integrated values from the fitted Gaussian and the integrated values obtained from the Riemann integral of the data set for all 500 conformational energies for each model. The difference between the two was less than 1% for all the models. These differences indicate a good estimation for the integrated peak.

Finally, the energy in the peak of each Gaussian was compared to the energy computed by the GBMV method. Figures S15 and S16 demonstrate similar relative conformational energies for all models in both methods.
