# Supplementary figures and images for: Interactions between Aβ and Mutated Tau Lead to Polymorphism and Induce Aggregation of Aβ-Mutated Tau Oligomeric Complexes
Source: PLoS One. 2013 Aug 12;8(8):e73303. doi: 10.1371/journal.pone.0073303 (PMC3741189; doi:10.1371/journal.pone.0073303)

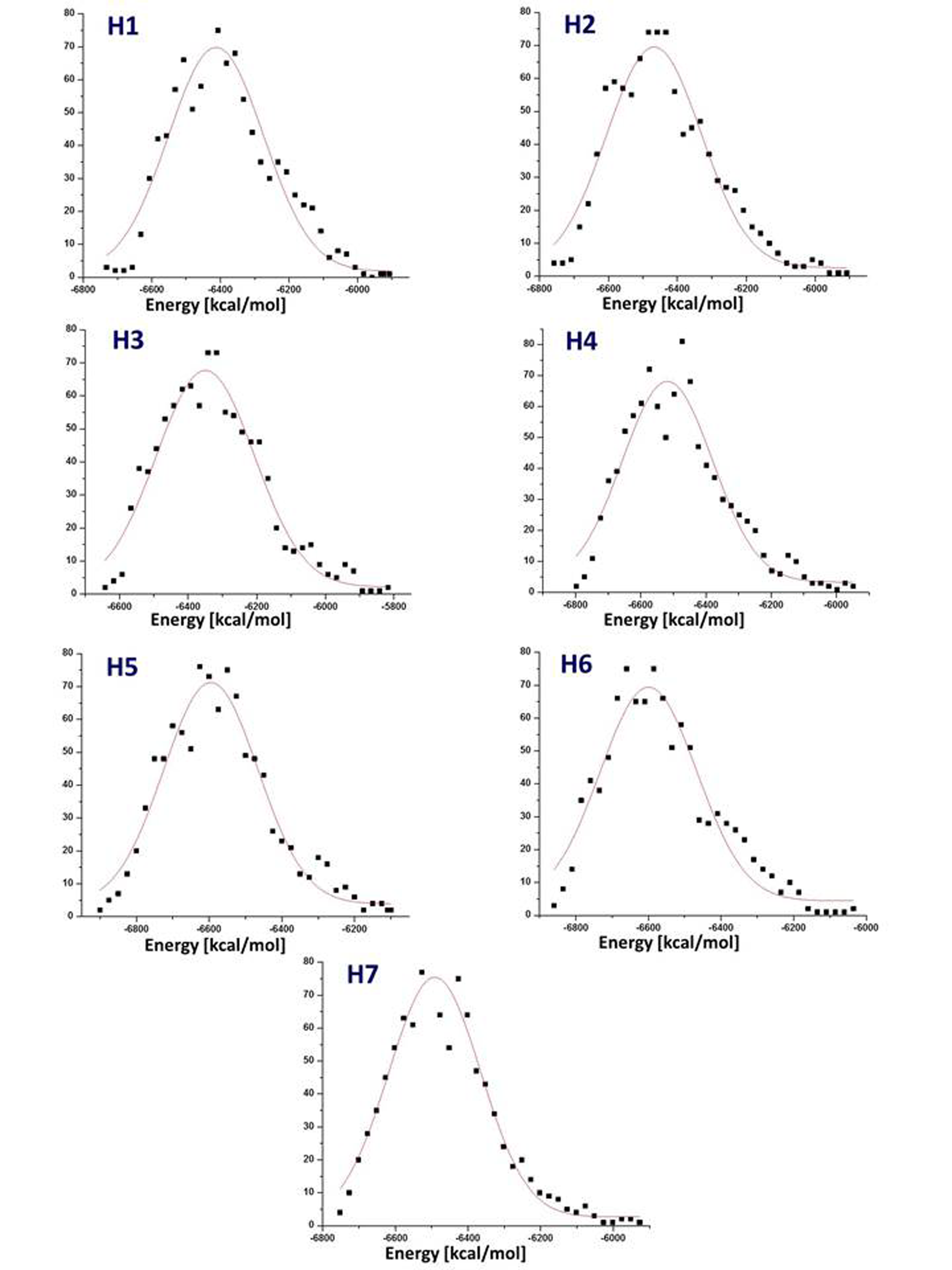

Supplement: Figure S1 — Scatter charts of the 500 conformations for H1-H4: obtained from the GBMV energy values extracted from the last 5 ns of each model: H1-H4 (black squares). The scatter charts represent the “histograms” of the number of conformations in energies’ ranges. The fitted curves (red line) were computed directly by Origin Pro 8. (TIF) [file pone.0073303.s002.tif]

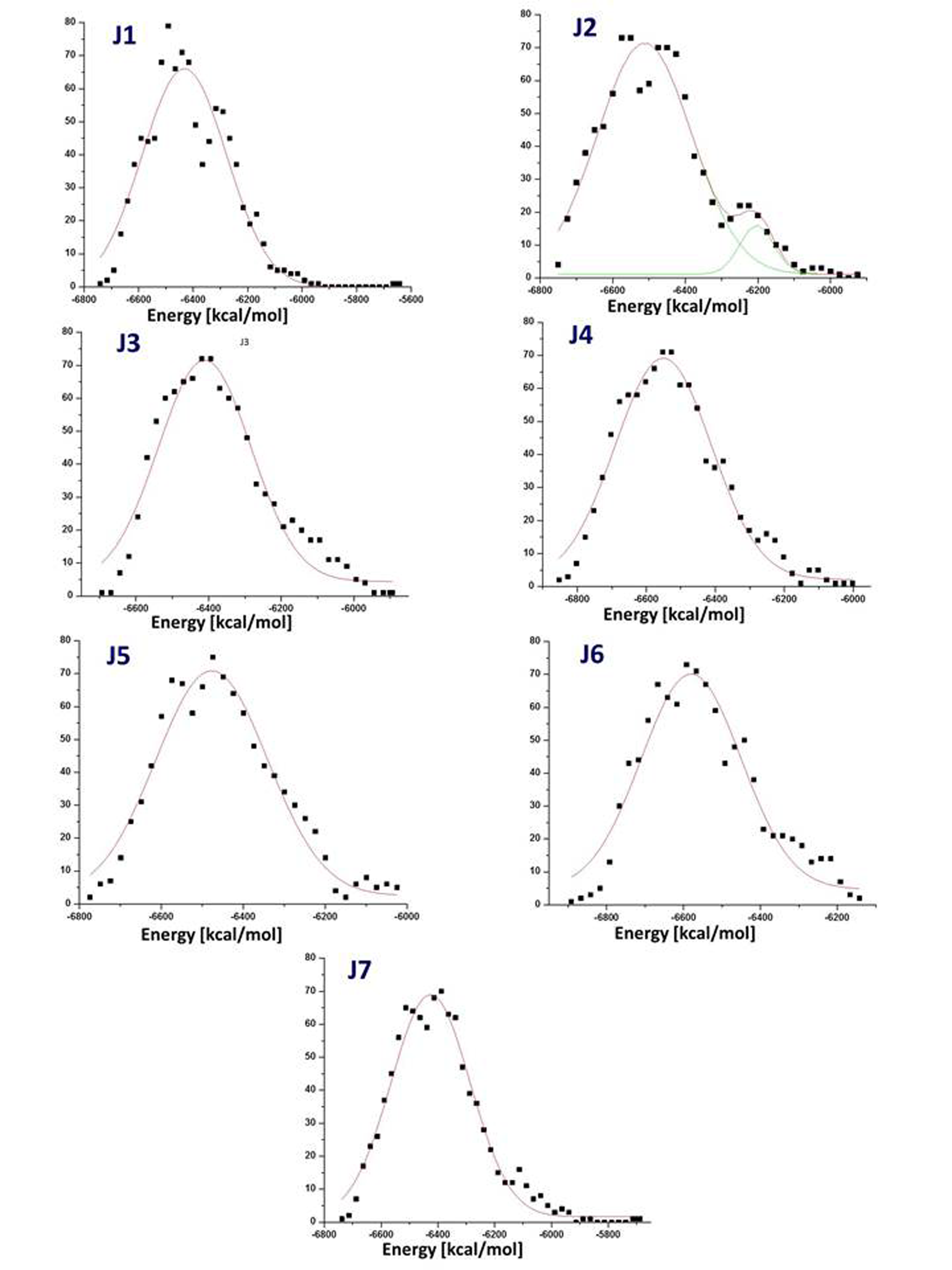

Supplement: Figure S2 — Scatter charts of the 500 conformations for J1-J4: obtained from the GBMV energy values extracted from the last 5 ns of each model: J1-J4 (black squares). The scatter charts represent the “histograms” of the number of conformations in the energy ranges. The fitted curves (red line) were computed directly by Origin Pro 8. (TIF) [file pone.0073303.s003.tif]

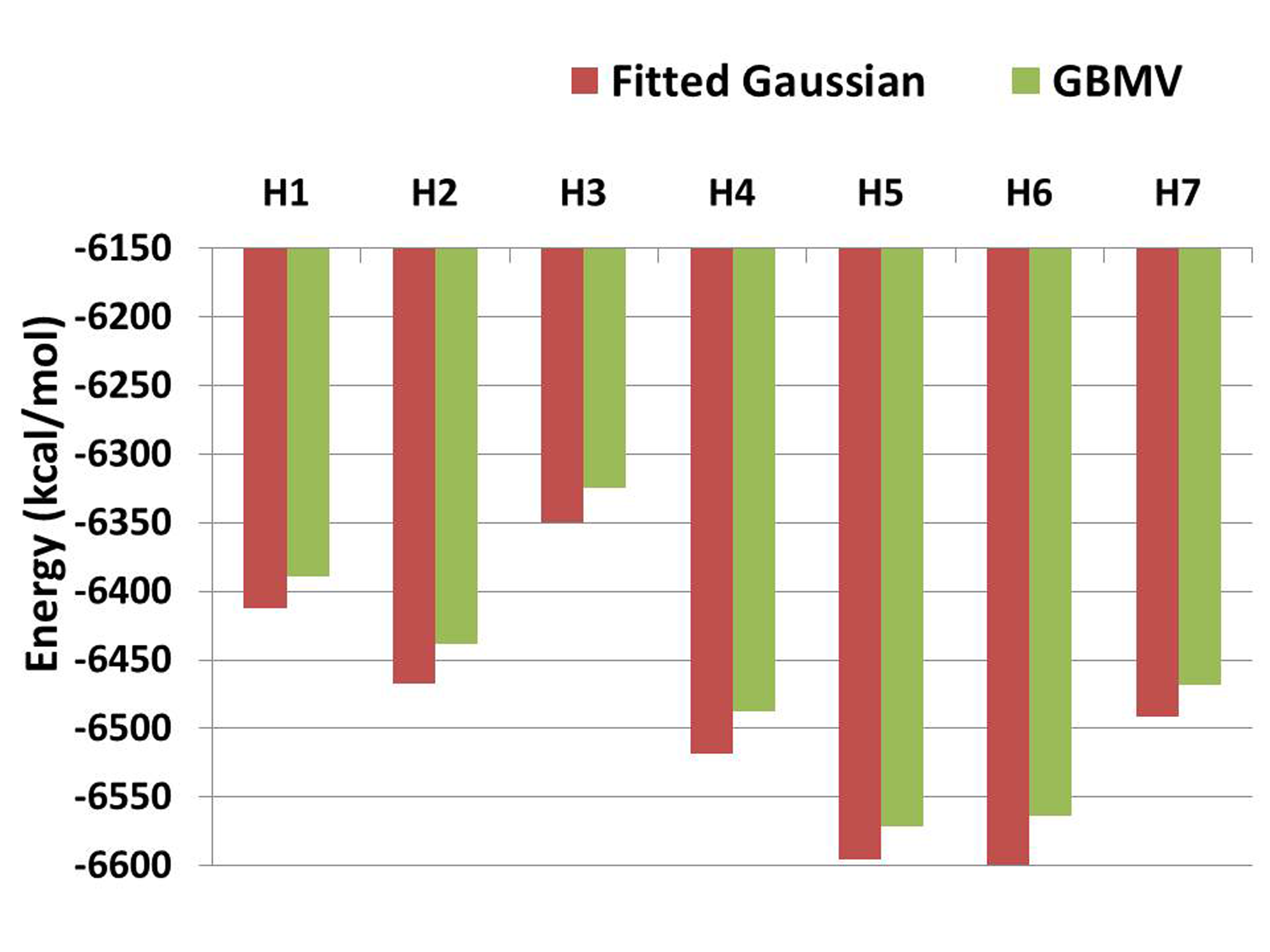

Supplement: Figure S3 — Comparison between GBMV and fitted Gaussian for H1-H7: Comparison between the averaged energies of the 500 conformations using the GBMV method and the energies obtained from the primary peaks of models H1-H7 using the peak fitting function of the origin with standard deviations (Figure S13). (TIF) [file pone.0073303.s004.tif]

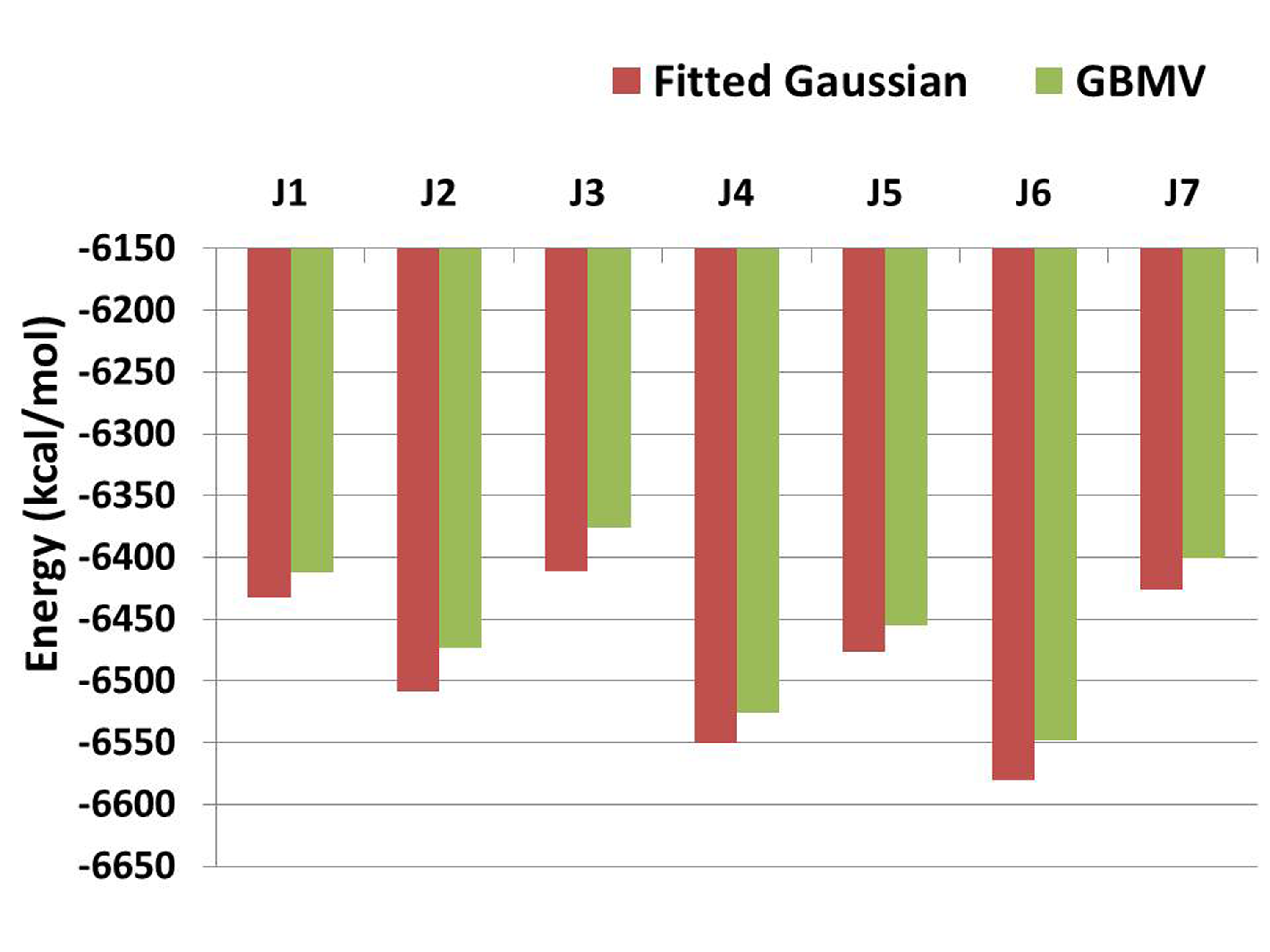

Supplement: Figure S4 — Comparison between GBMV and fitted Gaussian for J1-J7: Comparison between the averaged energies of the 500 conformations using the GBMV method and the energies obtained from the primary peaks of models J1-J7 using the peak fitting function of the origin with standard deviations (Figure S14). (TIF) [file pone.0073303.s005.tif]

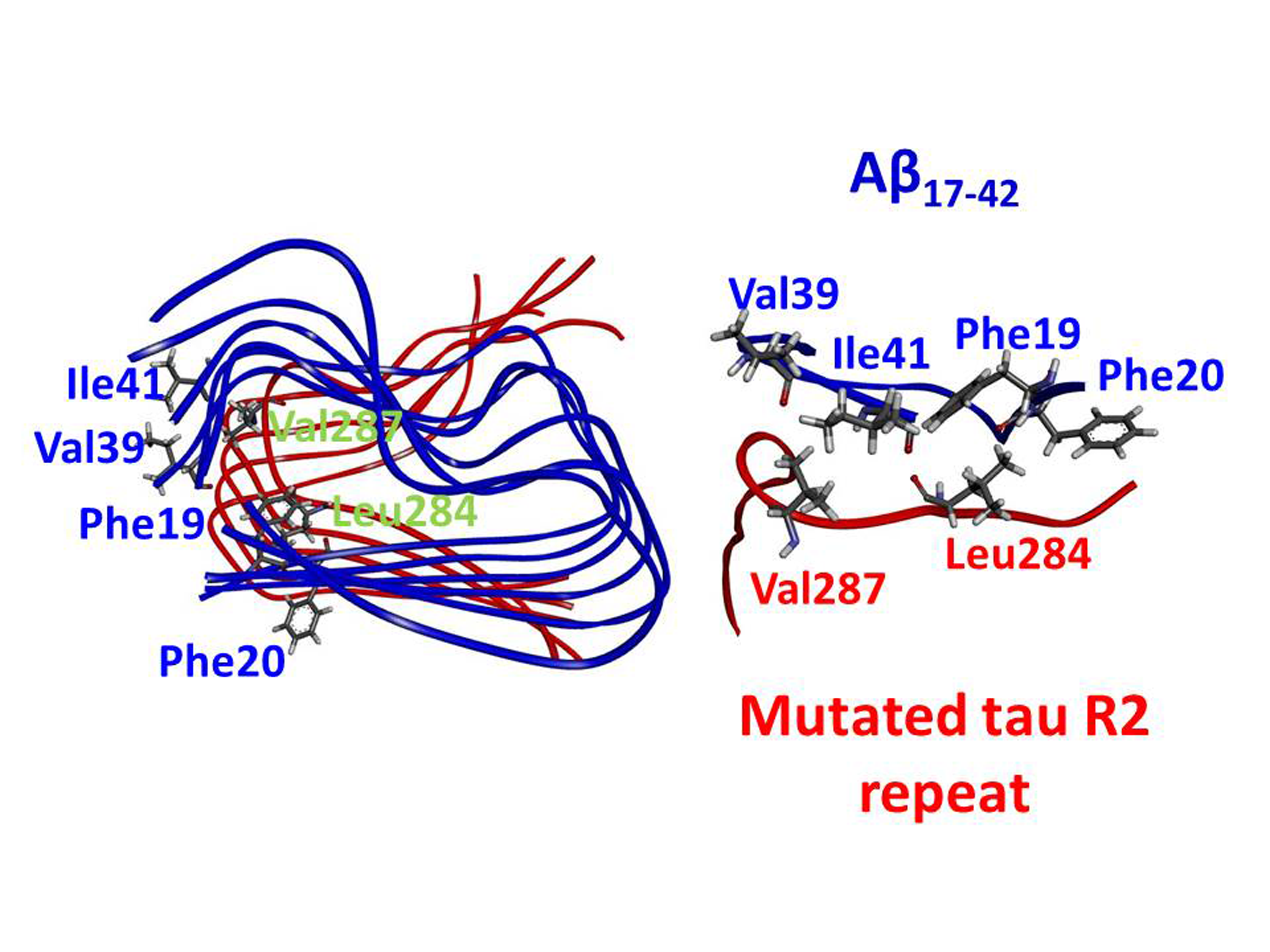

Supplement: Figure S5 — Hydrophobic interactions in model M2: Model J2 constitutes a relatively high portion of the total population in comparison with the other single-layer conformations, probably due to the hydrophobic interactions between Aβ17-42 (blue) and mutated tau (red) oligomers, which stabilized the structure. (TIF) [file pone.0073303.s006.tif]

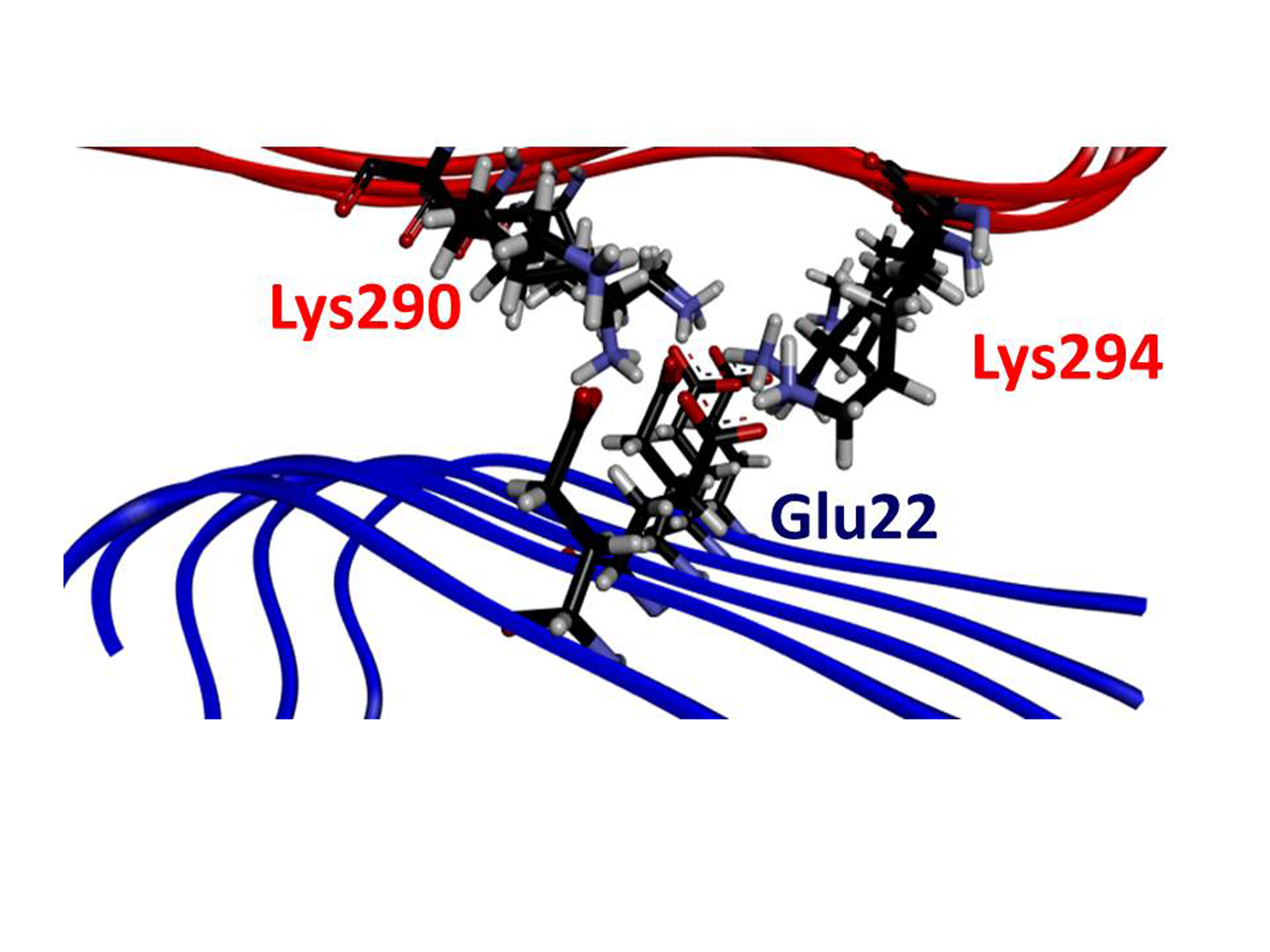

Supplement: Figure S6 — Salt-bridge interactions in model H5: The double-layer conformation of model H5 is stabilized by salt-bridge interactions in the interface region between Aβ17-42 (blue) and mutated tau (red) oligomers. (TIF) [file pone.0073303.s007.tif]

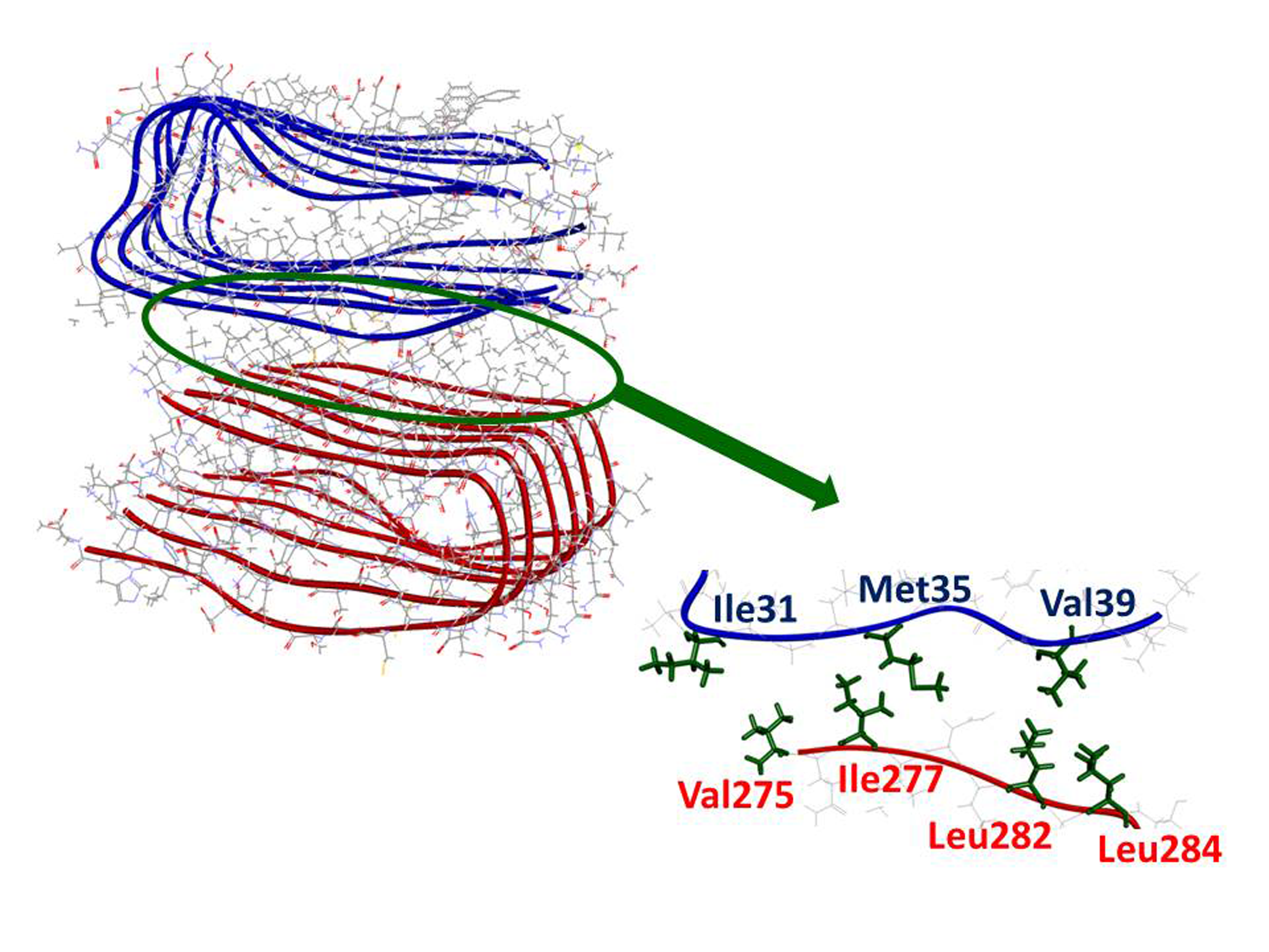

Supplement: Figure S7 — Hydrophobic interactions in model J6: The double-layer conformation of model J6 is stabilized by hydrophobic interactions in the interface region between Aβ17-42 (blue) and mutated tau (red) oligomers. (TIF) [file pone.0073303.s008.tif]

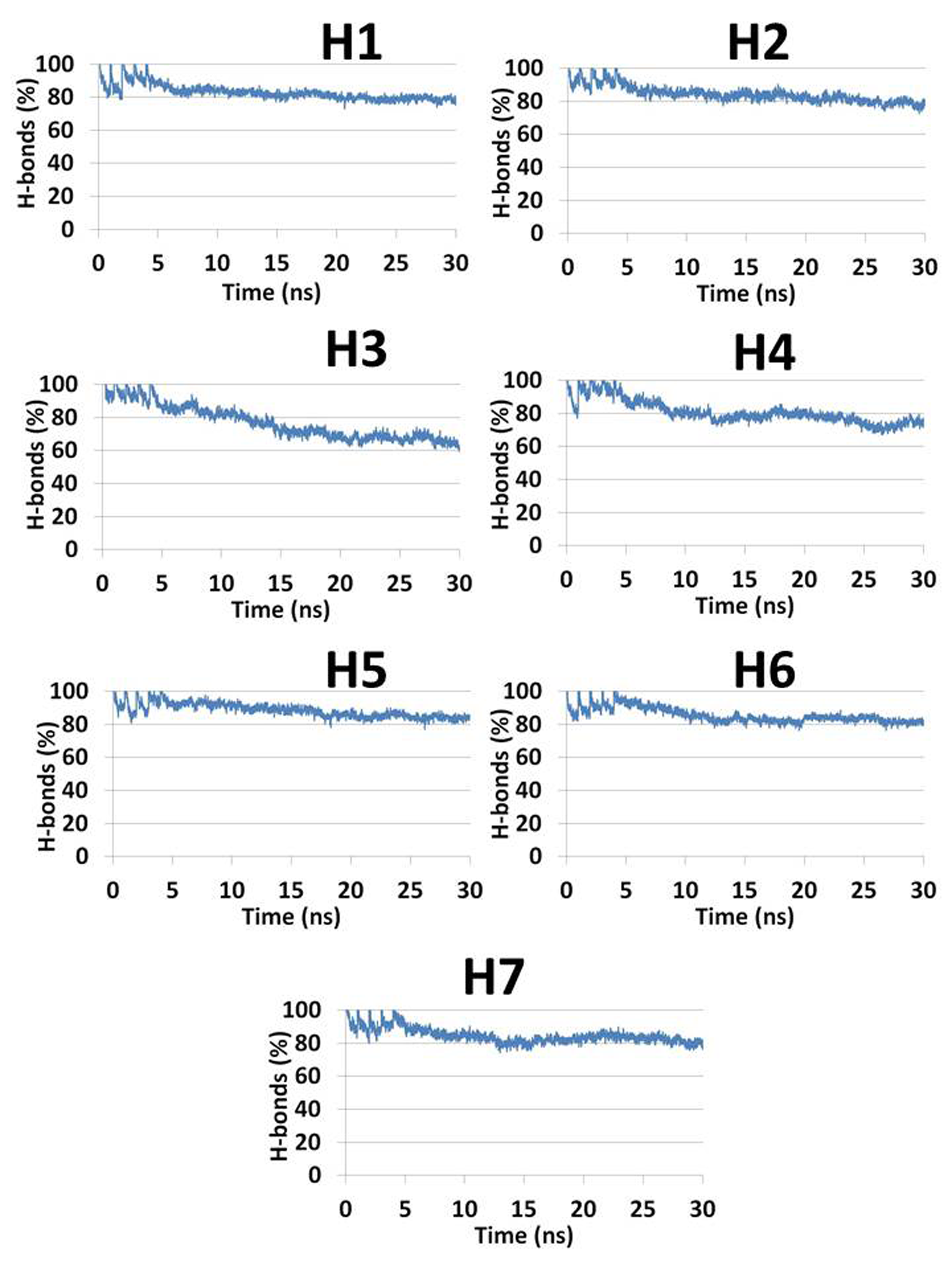

Supplement: Figure S8 — The fraction of the number of hydrogen bonds in models H1-H7: The fraction of the number of hydrogen bonds (in percentage) between all β-strands compared to the number in the initial oligomer for models H1-H7. (TIF) [file pone.0073303.s009.tif]

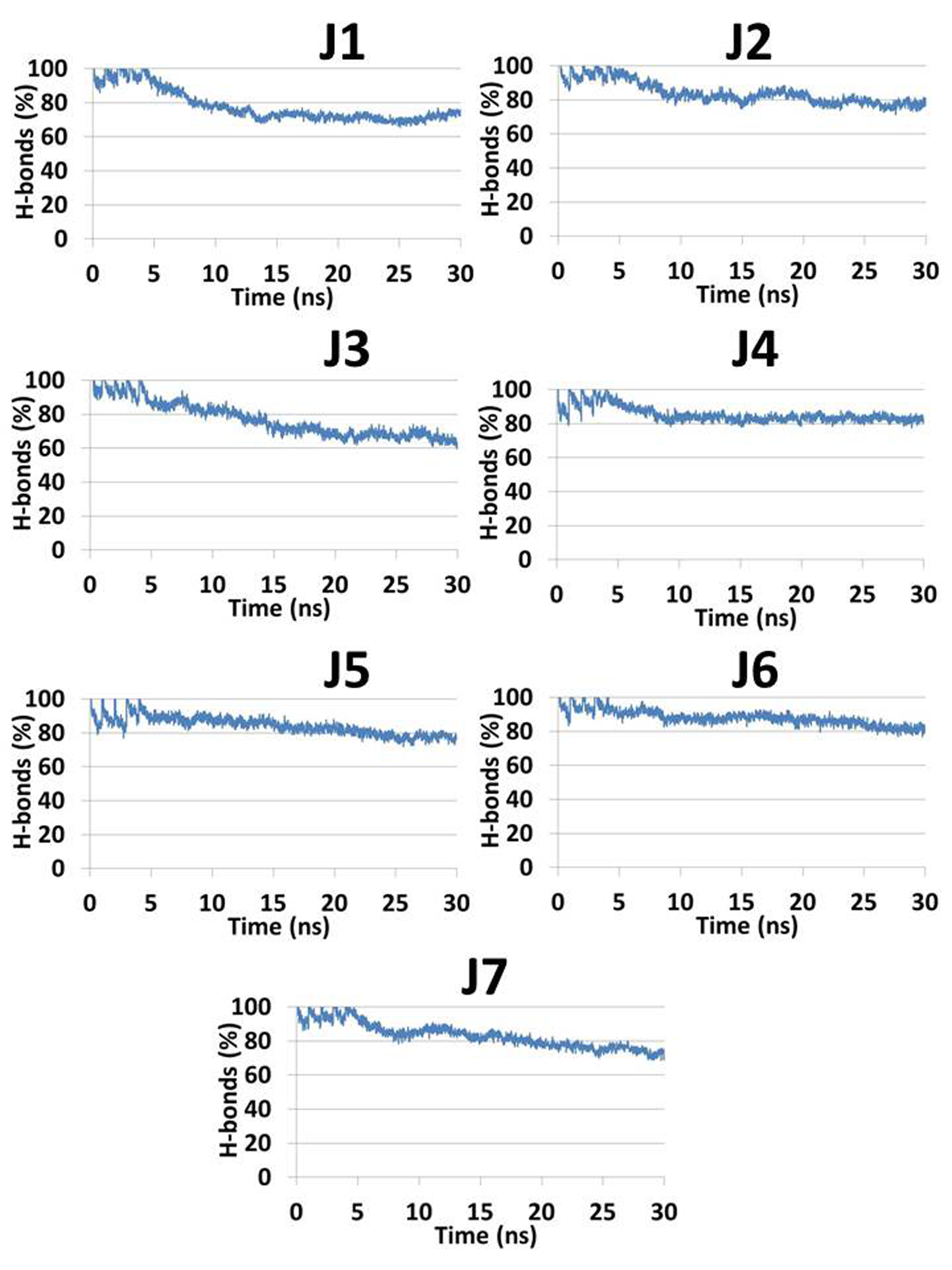

Supplement: Figure S9 — The fraction of the number of hydrogen bonds in models J1-J7: The fraction of the number of hydrogen bonds (in percentage) between all β-strands compare to the number in the initial oligomer for models J1-J7. (TIF) [file pone.0073303.s010.tif]

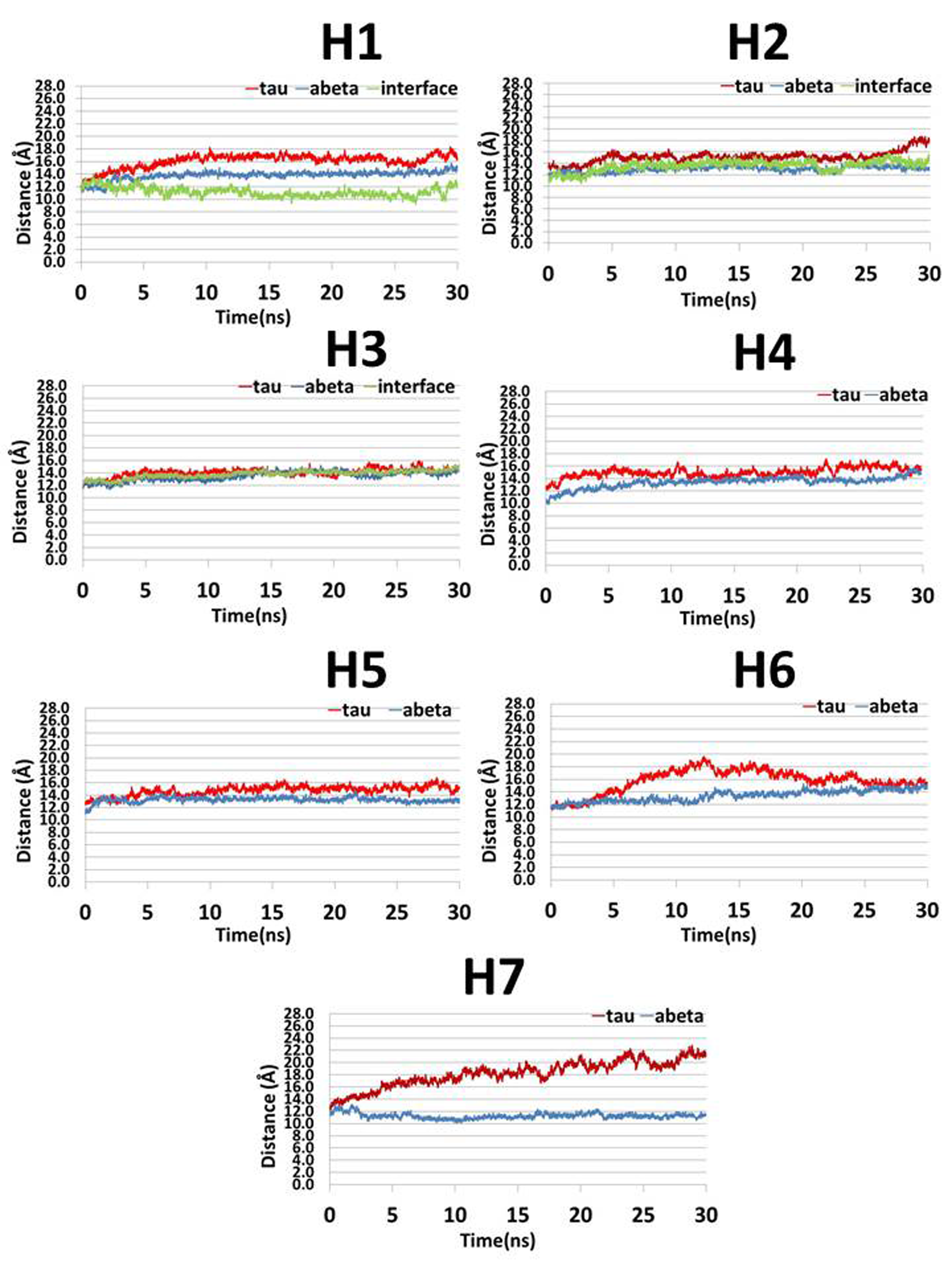

Supplement: Figure S10 — The averaged inter-sheet (Cα backbone-backbone) distances for models H1-H7 along the molecular dynamics (MD) simulations. (TIF) [file pone.0073303.s011.tif]

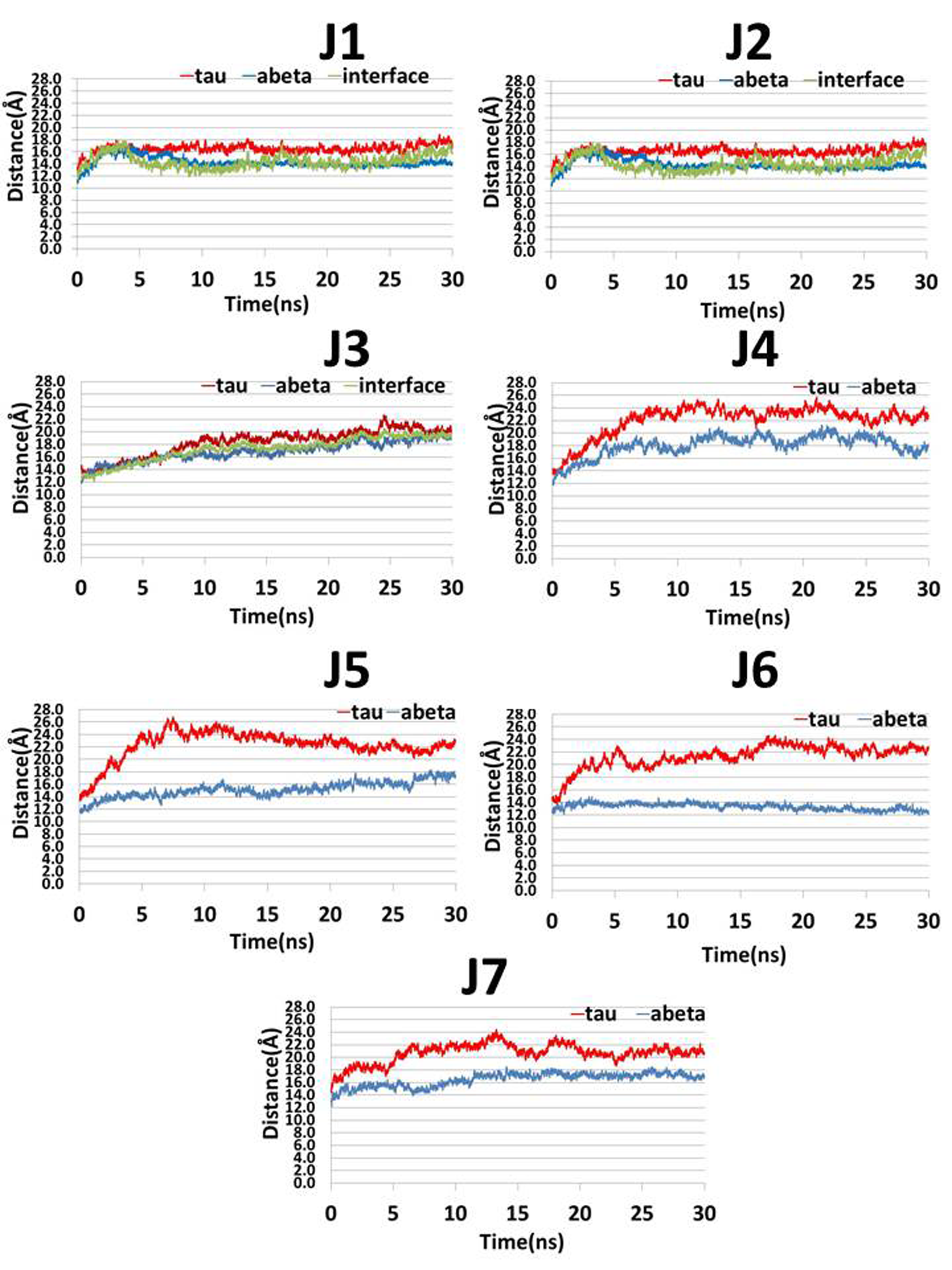

Supplement: Figure S11 — The averaged inter-sheet (Cα backbone-backbone) distances for models J1-J7 along the molecular dynamics (MD) simulations. (TIF) [file pone.0073303.s012.tif]

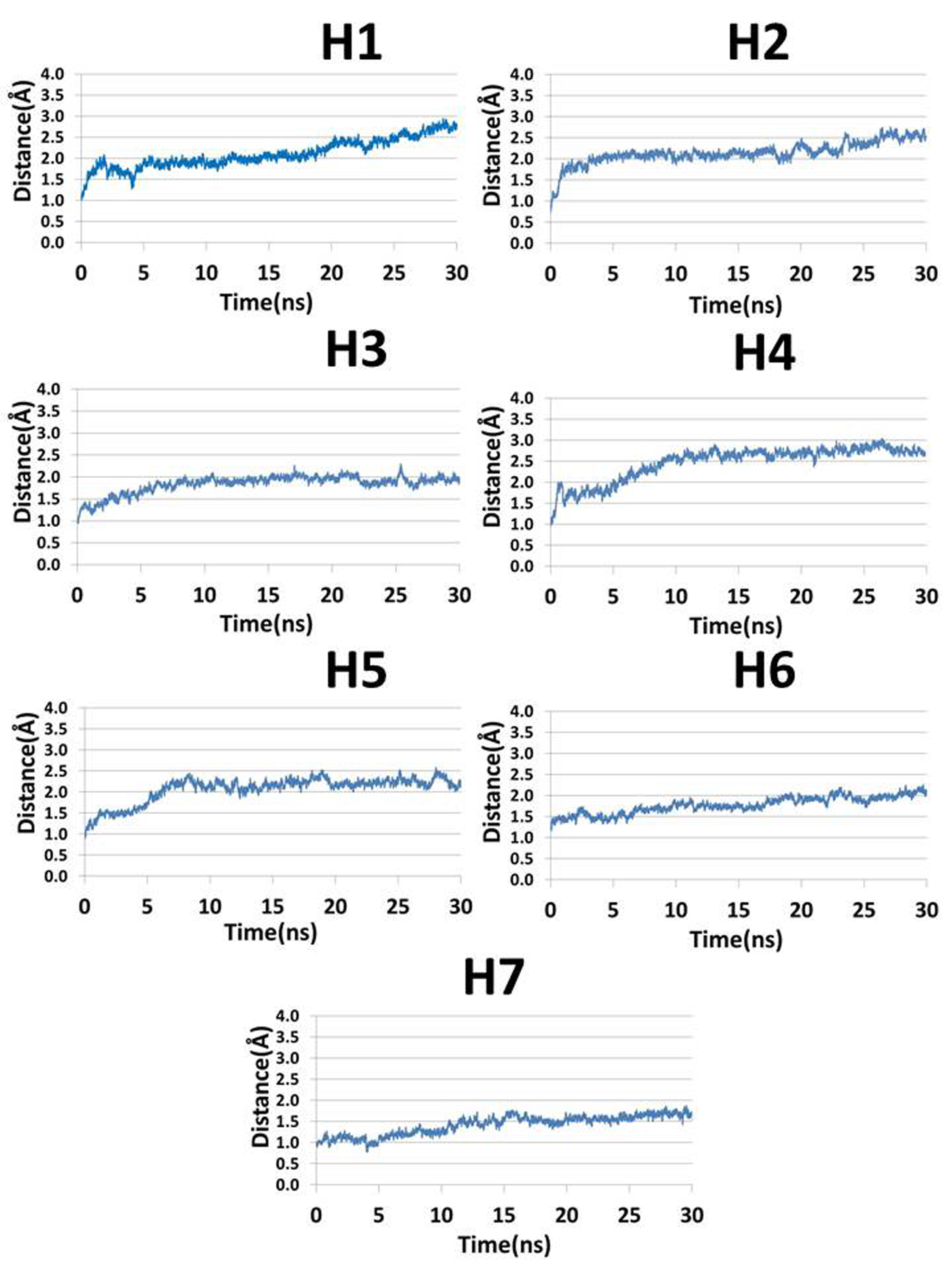

Supplement: Figure S12 — RMSDs of H1-H7. (TIF) [file pone.0073303.s013.tif]

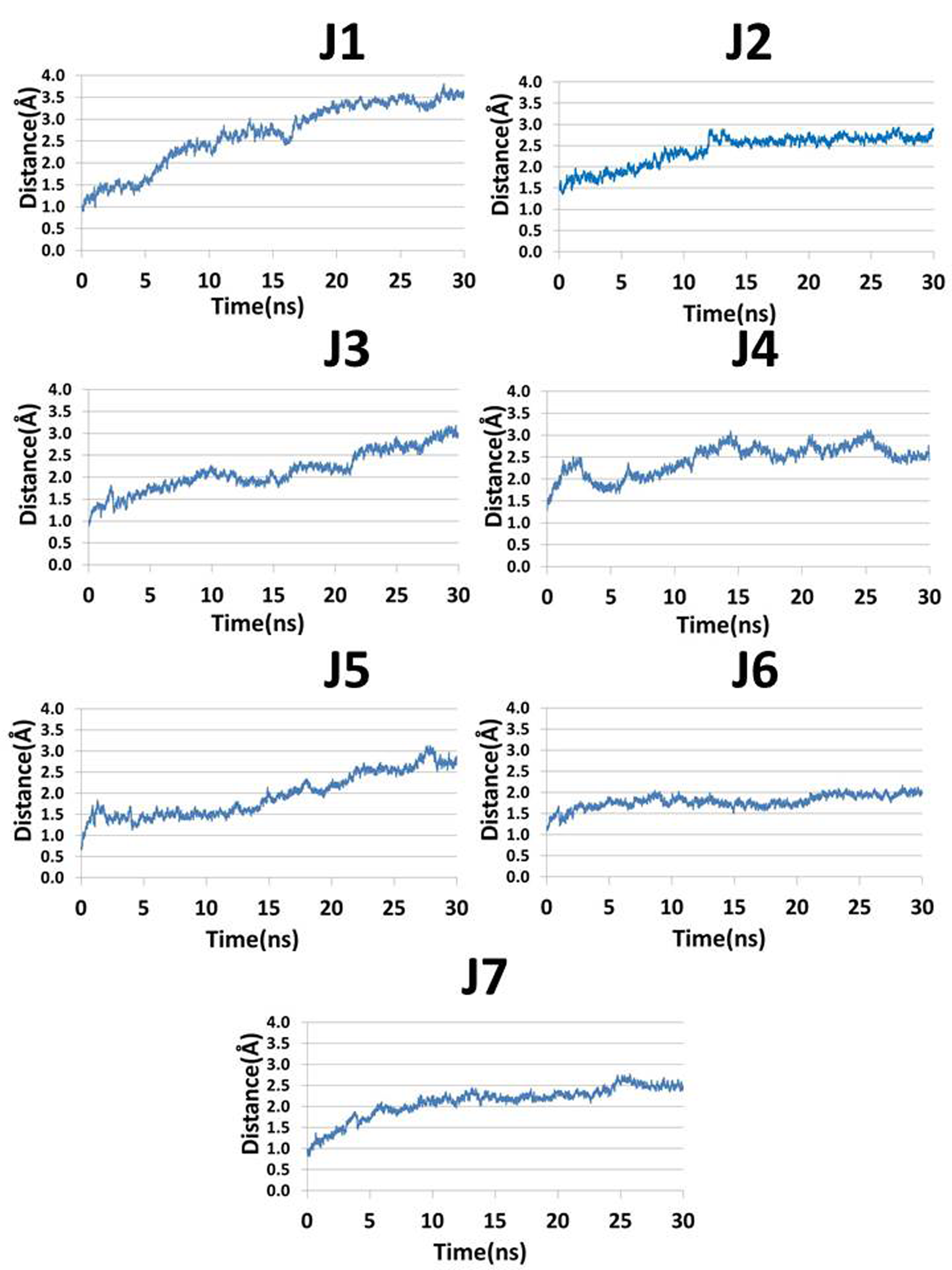

Supplement: Figure S13 — RMSDs of J1-J7. (TIF) [file pone.0073303.s014.tif]

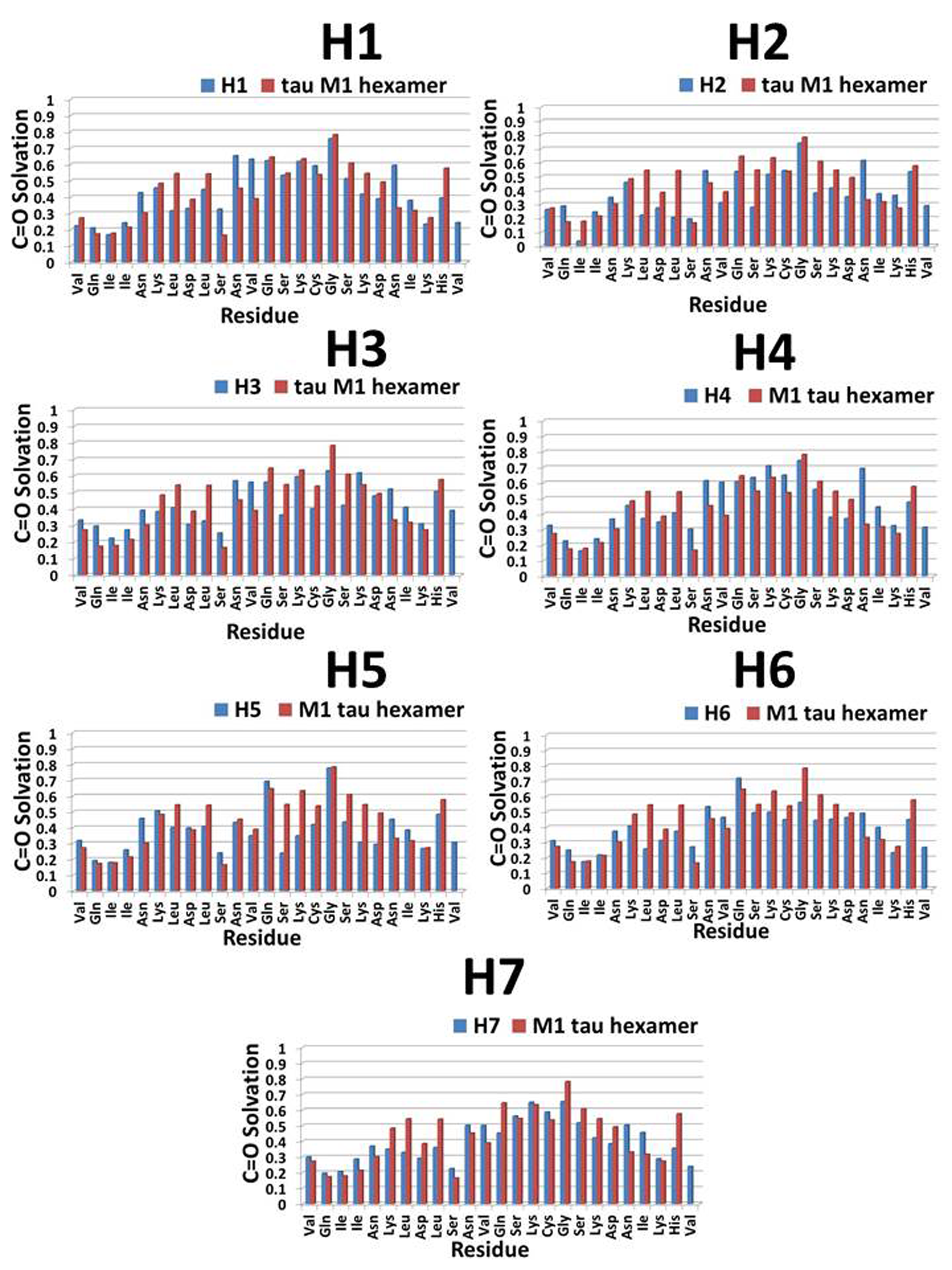

Supplement: Figure S14 — The average number of water molecules around each side chain Cβ carbon (within 4 Å) for models H1-H7. (TIF) [file pone.0073303.s015.tif]

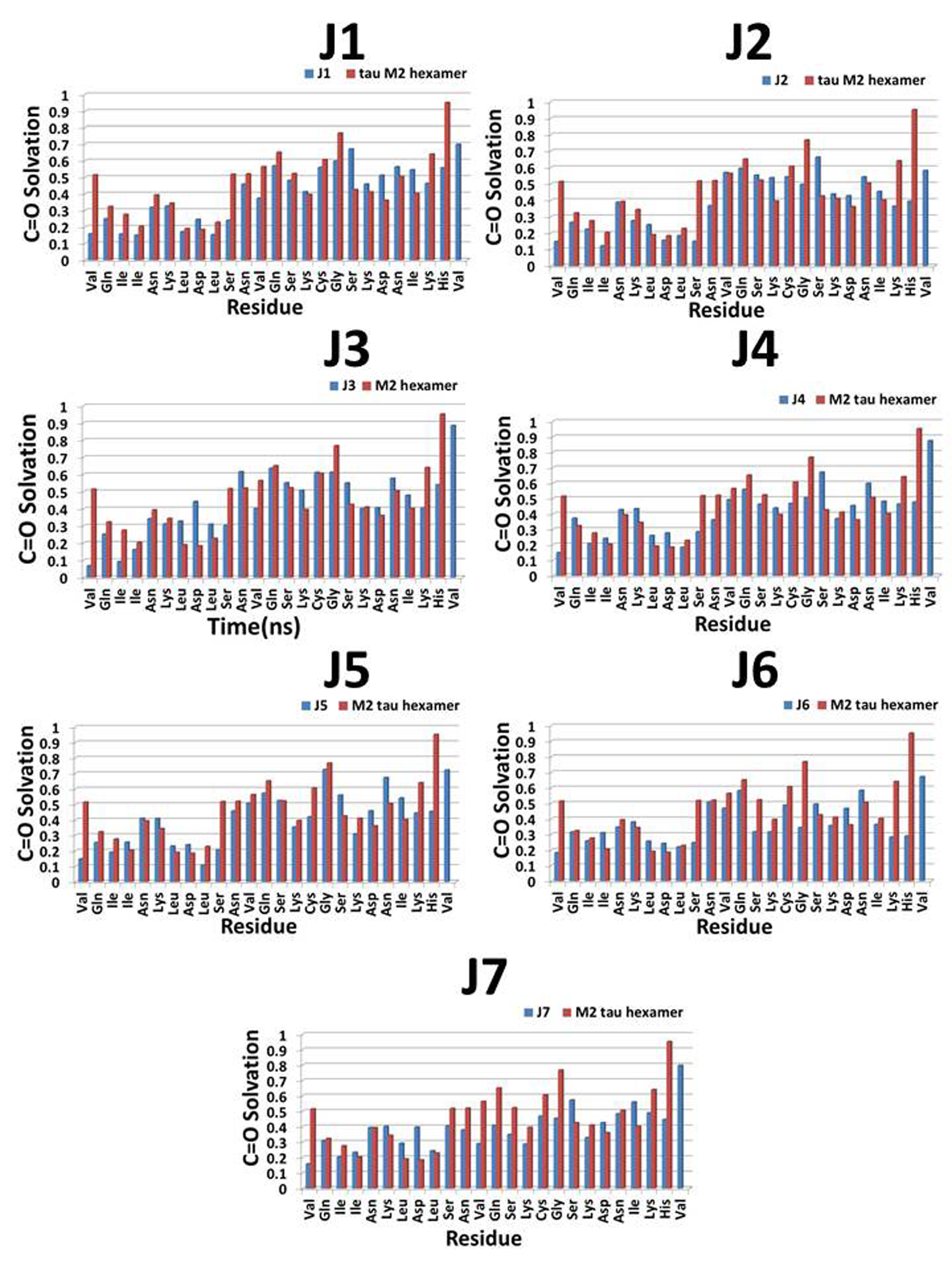

Supplement: Figure S15 — The average number of water molecules around each side chain Cβ carbon (within 4 Å) for models J1-J7. (TIF) [file pone.0073303.s016.tif]

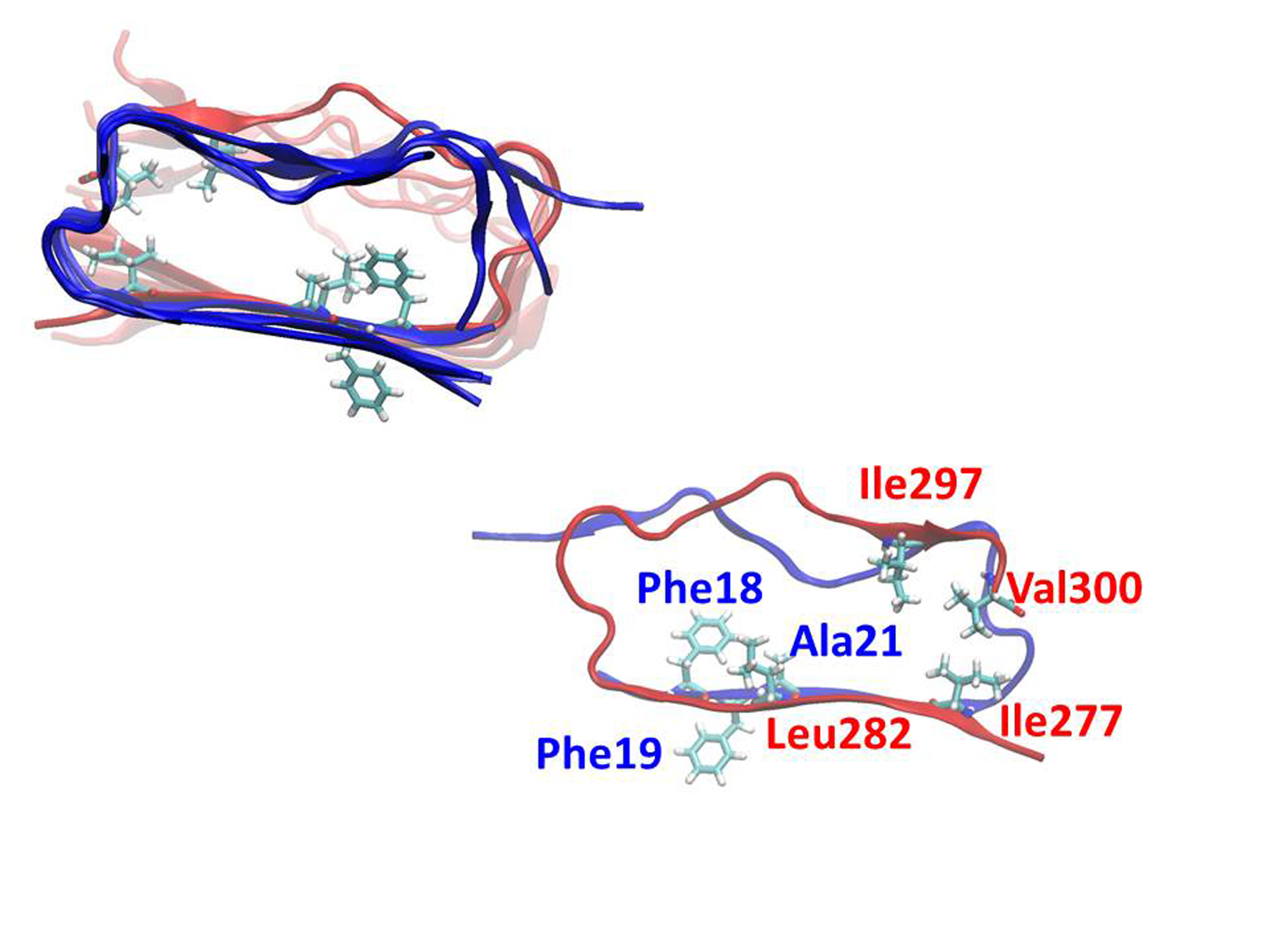

Supplement: Figure S16 — Hydrophobic interactions in model H2: The single-layer conformation of model H2 illustrates intramolecular hydrophobic interactions between Ile277, Ile297 and Leu300 of mutated tau, and intermolecular hydrophobic interactions between Leu288 of mutated tau and Ala21 of Aβ17-42. (TIF) [file pone.0073303.s017.tif]
