# Supplementary material for: Interactions between Aβ and Mutated Tau Lead to Polymorphism and Induce Aggregation of Aβ-Mutated Tau Oligomeric Complexes
Source: PLoS One. 2013 Aug 12;8(8):e73303. doi: 10.1371/journal.pone.0073303 (PMC3741189; doi:10.1371/journal.pone.0073303)
Supplement: Table S1 — The conformational energies (computed using the GBMV calculations) and the populations of models H1-H7 and model J1-J7. (PDF) [file pone.0073303.s018.pdf]

Table S1: The conformational energies (computed using the GBMV calculations) and the populations of models H1-H7 and model J1-J7.

**Model**	**Conformational energy (kcal/mol)**	**Population (%)**	**Standard deviation (kcal/mol)**
J1	-6412.25	6.4	147.02
J2	-6472.78	7.5	150.17
J3	-6375.49	5.0	146.39
J4	-6526.12	8.8	147.31
J5	-6455.26	6.9	141.90
J6	-6547.89	9.4	148.53
J7	-6400.71	5.6	153.30
H1	-6389.40	5.2	142.40
H2	-6438.53	6.6	148.66
H3	-6324.98	3.8	151.30
H4	-6487.32	7.3	153.85
H5	-6571.71	9.9	147.76
H6	-6564.15	9.8	150.53
H7	-6468.06	7.9	140.22
