# Supplementary material for: Interactions between Aβ and Mutated Tau Lead to Polymorphism and Induce Aggregation of Aβ-Mutated Tau Oligomeric Complexes
Source: PLoS One. 2013 Aug 12;8(8):e73303. doi: 10.1371/journal.pone.0073303 (PMC3741189; doi:10.1371/journal.pone.0073303)
Supplement: Table S2 — The averaged inter-sheet (Cα backbone-backbone) distances measured in the last 5 ns of the MD simulations for models H1-H7 and models J1-J7. (PDF) [file pone.0073303.s019.pdf]

Table S2: The averaged inter-sheet (Cα backbone-backbone) distances measured in the last 5 ns of the MD simulations for models H1-H7 and models J1-J7.

**Model**	**Tau backbone-backbone distance [Å]**	**Aβ backbone-backbone distance [Å]**	**Aβ-Tau backbone-backbone distance [Å]**
J1	19.6	13.3	17.2
J2	17.1	14.0	15.4
J3	20.5	19.0	19.4
J4	22.5	18.1	-
J5	21.8	16.8	-
J6	22.3	12.7	-
J7	21.0	17.3	-
H1	16.4	14.4	11.1
H2	16.4	13.3	14.3
H3	14.5	14.1	14.4
H4	15.8	14.2	-
H5	15.3	13.0	-
H6	15.3	14.5	-
H7	20.5	11.2	-
