# Supplementary material for: Interactions between Aβ and Mutated Tau Lead to Polymorphism and Induce Aggregation of Aβ-Mutated Tau Oligomeric Complexes
Source: PLoS One. 2013 Aug 12;8(8):e73303. doi: 10.1371/journal.pone.0073303 (PMC3741189; doi:10.1371/journal.pone.0073303)
Supplement: Table S3 — The conformational energies (computed using the GBMV calculations) and the populations of wild-type (WT) TauR2 repeat oligomer, the mutated tau M1 and M2 models and Aβ17-42 oligomer. (PDF) [file pone.0073303.s020.pdf]

Table S3: The conformational energies (computed using the GBMV calculations) and the populations of wild-type (WT) Tau R2 repeat oligomer, the mutated tau M1 and M2 models and A$\beta_{17\text{-}42}$ oligomer.

Model	GBMV average energy [kcal/mol]	Standard Deviaton [kcal/mol]
**WT Tau R2 repeat**	-4881.49	99.85
**M1**	-4618.03	94.06
**M2**	-4349.05	87.18
**A$\beta_{17\text{-}42}$**	-1845.66	77.55
